# Supplementary material for: Efficient polygenic risk scores for biobank scale data by exploiting phenotypes from inferred relatives
Source: Nat Commun. 2020 Jun 17;11:3074. doi: 10.1038/s41467-020-16829-x (PMC7299943; doi:10.1038/s41467-020-16829-x)
Supplement: Supplementary file 1 — Supplementary information [file 41467_2020_16829_MOESM1_ESM.docx]

# **Efficient polygenic risk scores for biobank scale data by exploiting phenotypes from inferred relatives**

Truong et al.

**SUPPLEMENTARY FIGURES**


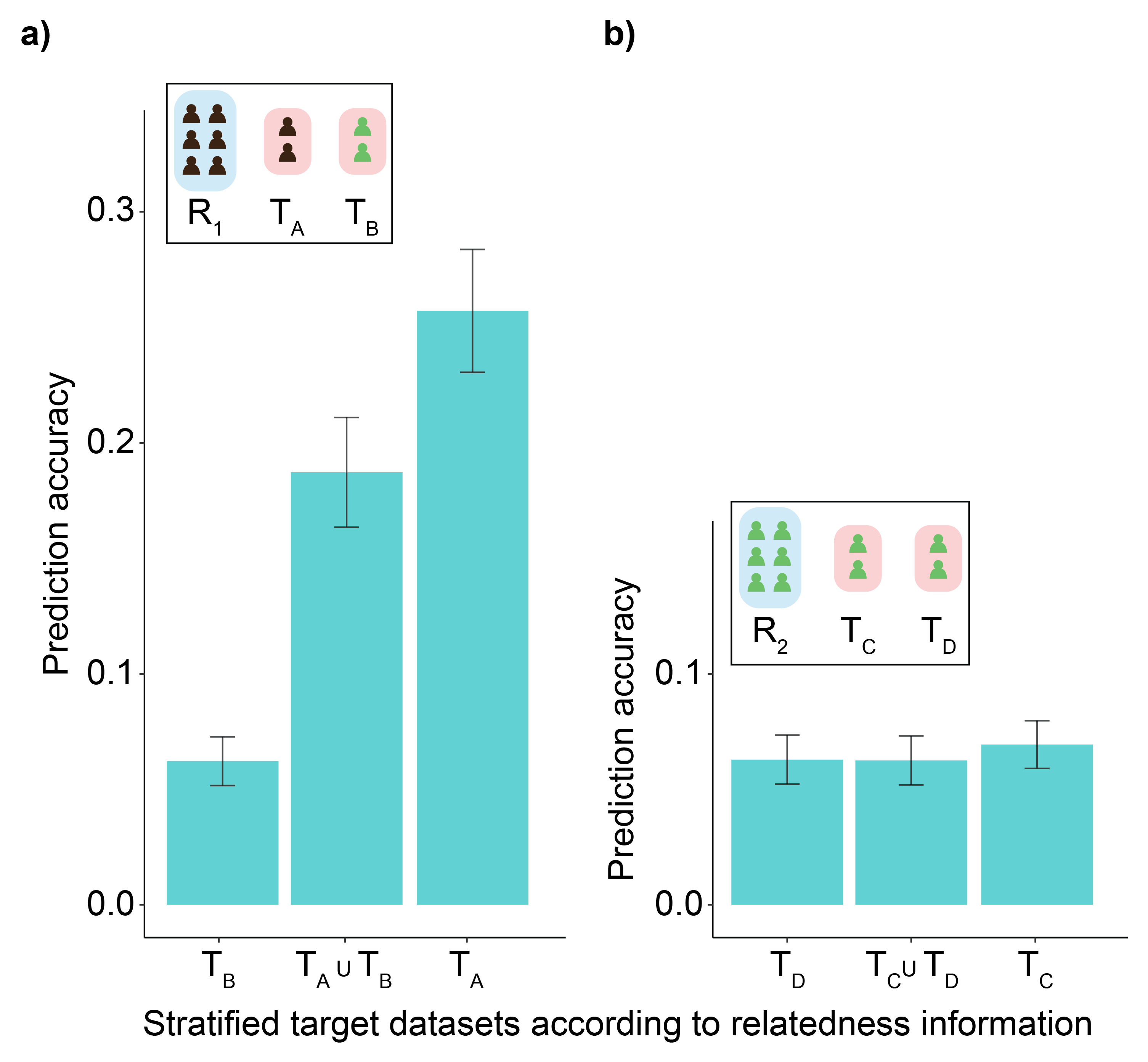


**Supplementary Figure 1. Prediction accuracies for target datasets stratified according to their relationships with the reference dataset.** In the prediction, 5000 reference and 2000 target samples were used, where T_A_ was a set of 1000 target samples that had 1^st^ degree relationships with the reference samples, and T_B_ was the other 1000 target samples that were unrelated to the reference samples (a). For a comparison, a separate analysis was considered, in which both target sets (T_C_ and T_D_) were unrelated to the reference samples (b). The main bars represent the mean values averaged over the analyses of 50 traits. The error-bars show the 95% confidence intervals of the mean values.


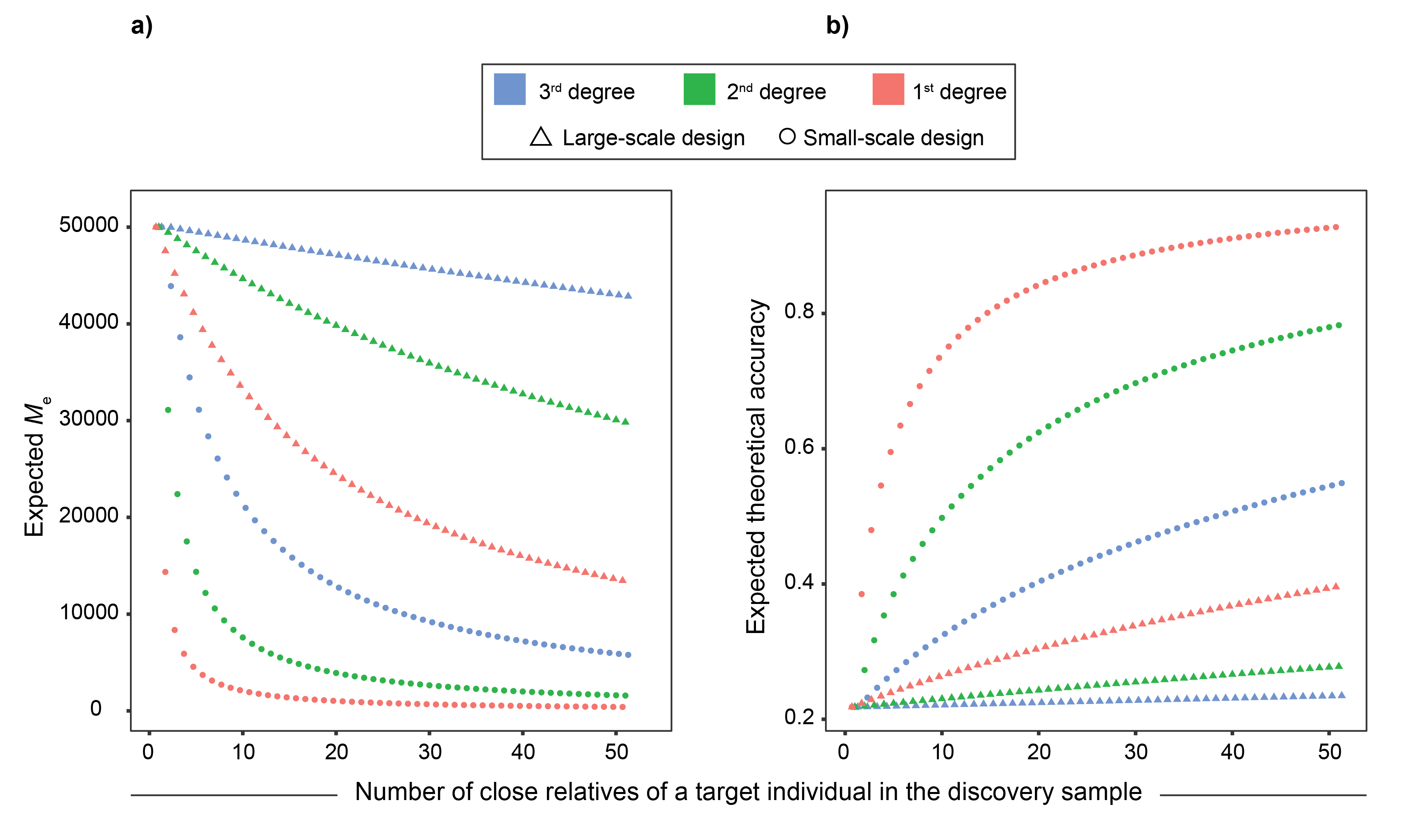


**Supplementary Figure 2. The effective number of chromosome segments (**$\boldsymbol{M}_{\boldsymbol{e}}$**) and prediction accuracy when varying the number of relatives of a target individual in the discovery sample.** Expected $M_{e}$ (a) and theoretical prediction accuracy (b) for a single target individual when adding a different number of his or her close relatives in the discovery dataset. Here, we analytically derived *M_e_* value of a single target individual when adding his or her close relatives (upto 50) in the discovery dataset of the large- and small-scale design (230,000 and 5,000 individuals that are unrelated to the target individual). When there is no relatives in the discovery dataset, the M_e_ value of the target individual is 50,000 as observed in Figure 2c. Based on these (genetically distant) relationships between the target individual and discovery sample, we added a relationship of 0.125, 0.25 or 0.5 when adding a 3^rd^, 2^nd^ or 1^st^ degree relative of the target individual in the discovery dataset. Then, we recalculated the variance of the relationships between the target individual and discovery sample now the relatives of the target individual. M_e_ value was obtained from the inverse of the variance of relationships. Theoretical prediction accuracy was calculated from Equation 1 given obtained M_e_ values, assuming a heritability of 0.5.


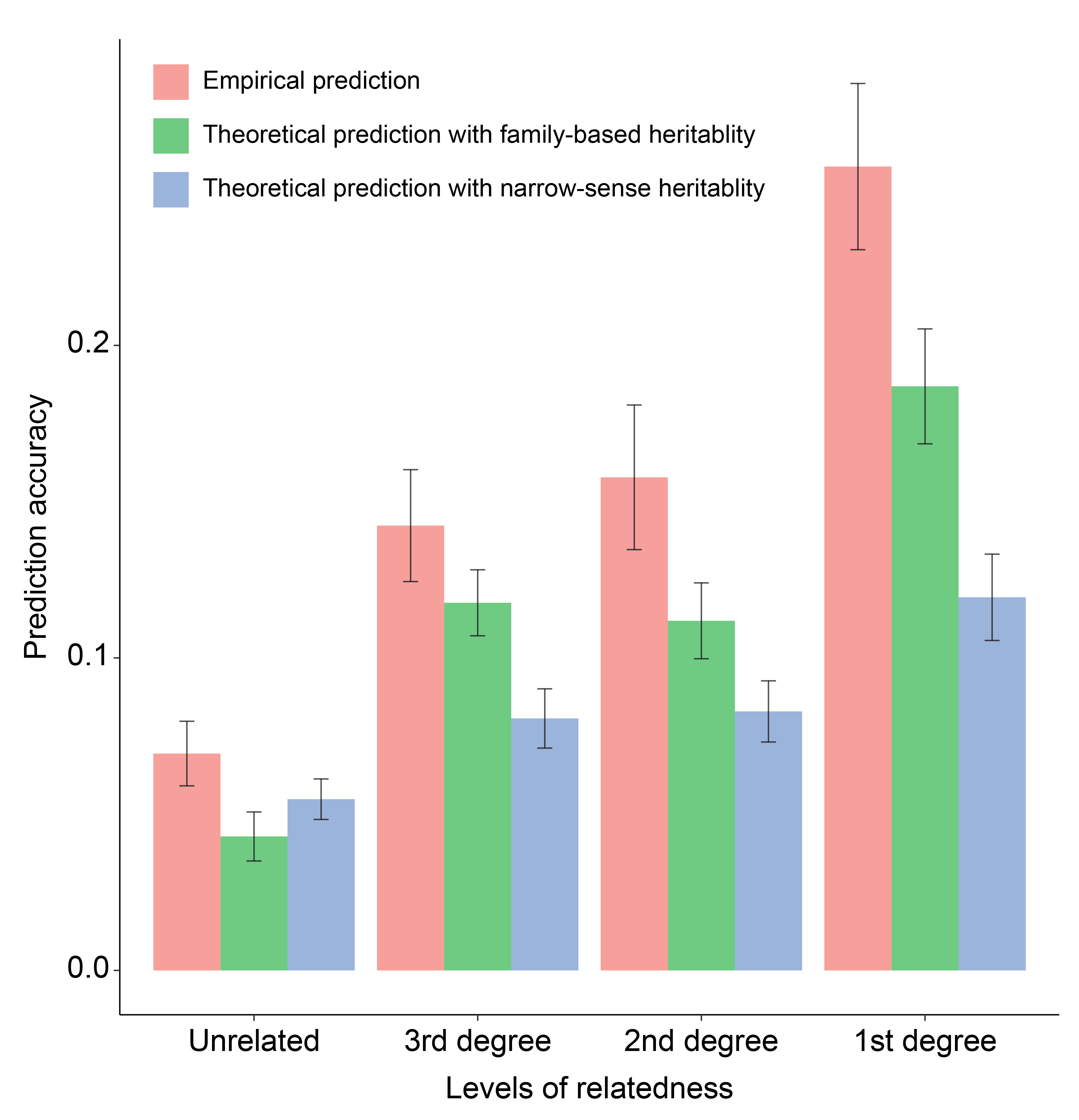


**Supplementary Figure 3. The actual empirical and theoretical prediction accuracies.** Theoretical prediction accuracies for analyses with unrelated, 3^rd^, 2^nd^ and 1^st^ degree relatives were calculated with family-based or narrow-sense heritability. It is noted that family-based heritability for unrelated individuals was estimated based on the small-scale design with unrelated samples. The main bars represent the mean values and the error-bars show the 95% confidence intervals of the mean values averaged over the analyses of 50 traits.


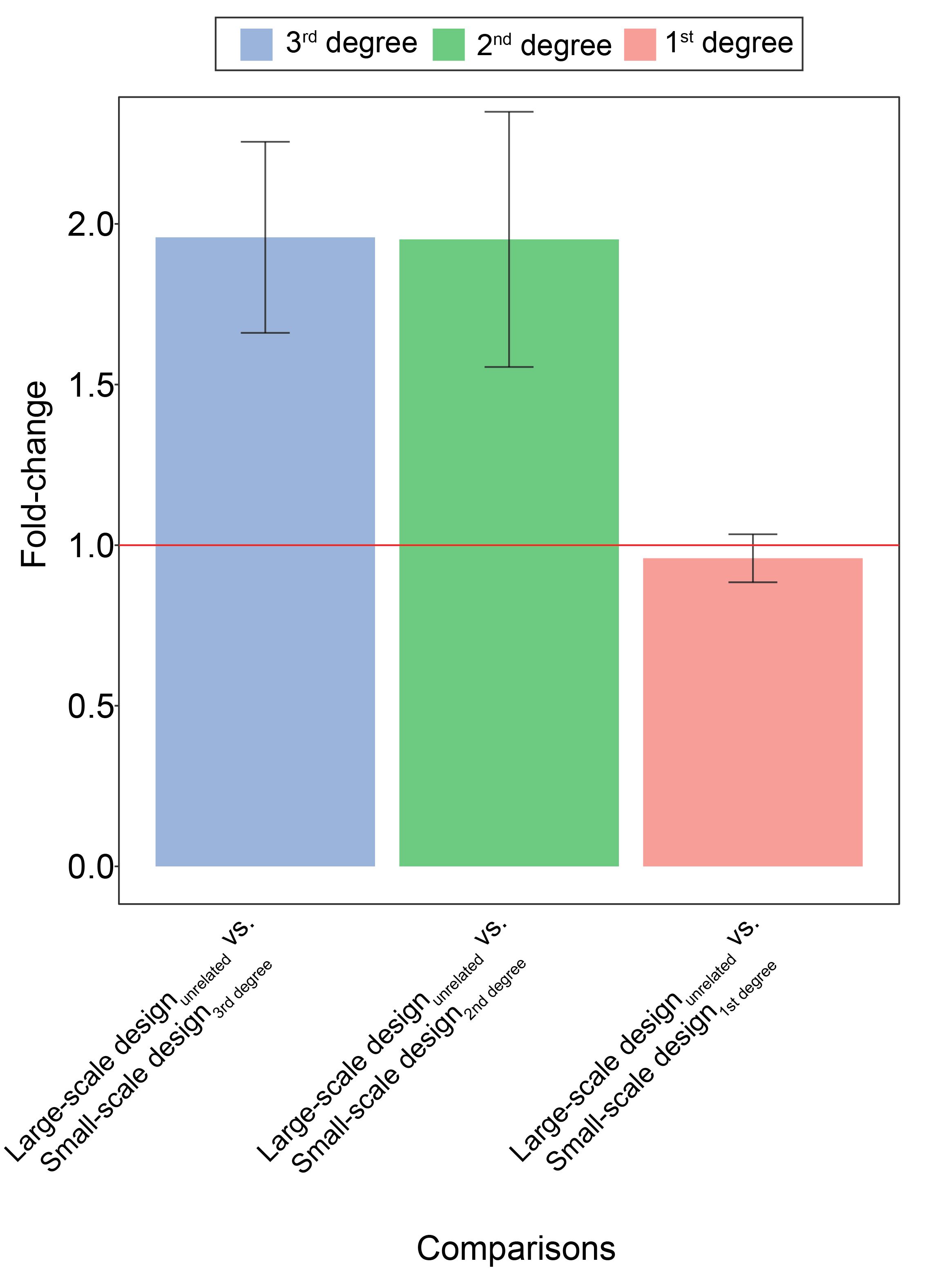


**Supplementary Figure 4. Comparisons of accuracies between polygenic prediction using unrelated sample in large-scale design against polygenic prediction using close relatives in small-scale design.** The main bars represent the mean fold-changes and the error-bars show the 95% confidence intervals of the mean fold-changes averaged over the analyses of 50 traits. The red horizontal line indicates ratio = 1.

**
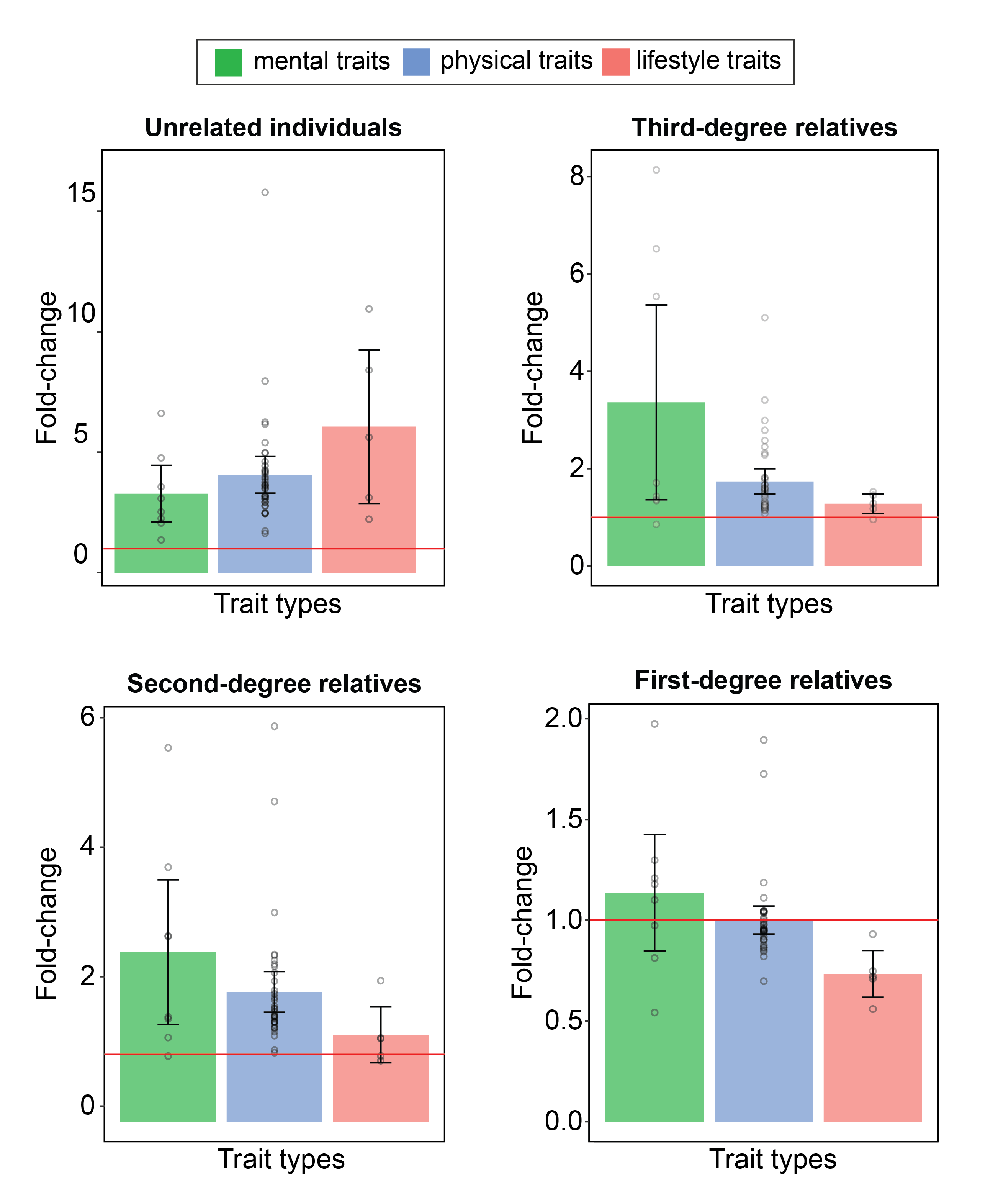
**

**Supplementary Figure 5. The ratio between the empirical prediction accuracies in the large- and small-scale designs for 3 types of traits.** The ratio of prediction accuracy in the large-scale design to the small-scale design with was compared across 3 different types of 50 traits, mental traits, physical traits and lifestyle traits. The main bars represent the mean fold-changes averaged over the analyses of 8, 37 and 5 traits in mental, physical and lifestyle traits, respectively. The error-bars show the 95% confidence intervals of the mean fold-changes. The red horizontal line indicates ratio = 1.

**
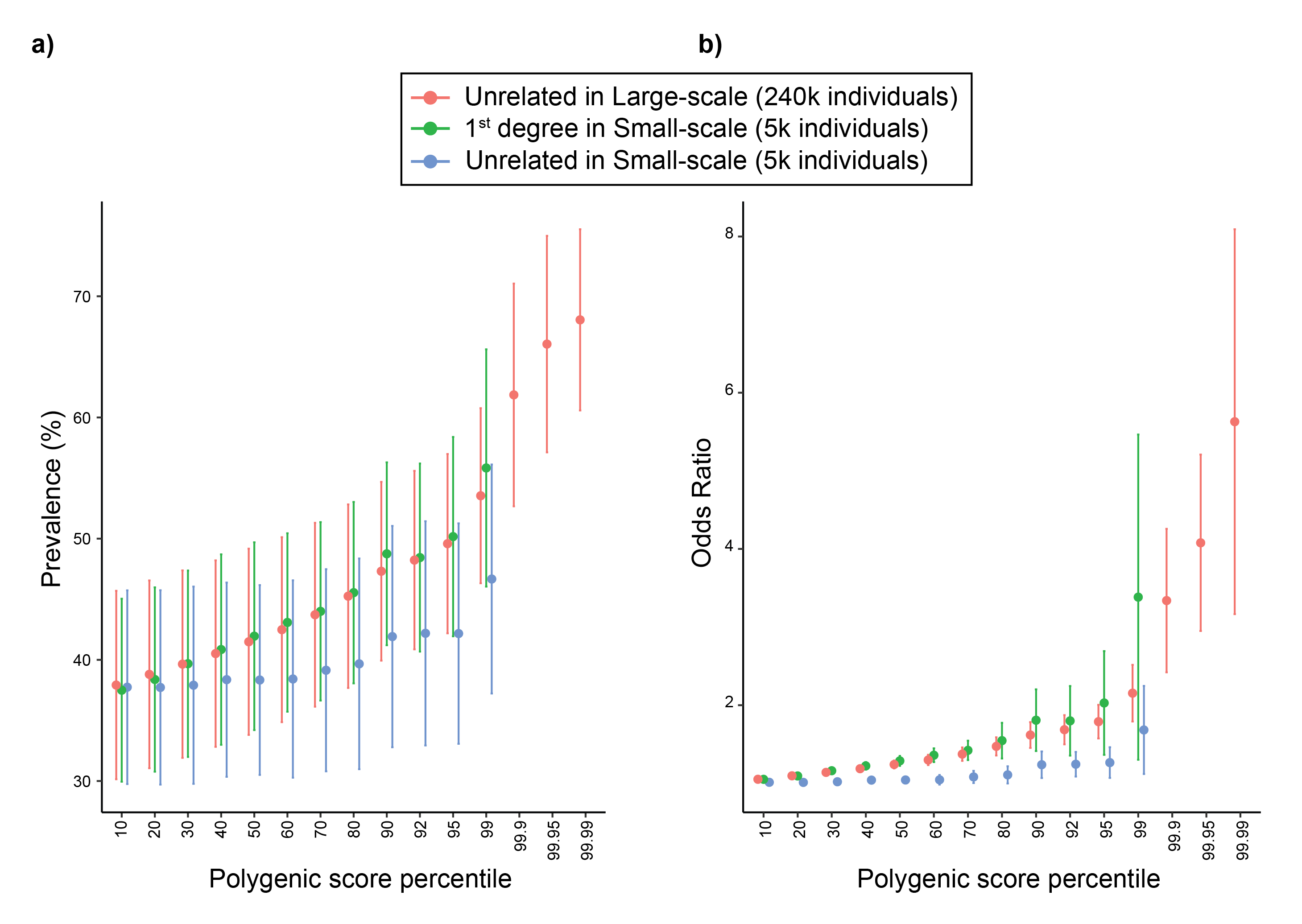
**

**Supplementary Figure 6. Risk for dichotomous traits according to polygenic risk score (PRS).** Prevalence (a) and odds ratio (b) by PRS binned above thresholds by percentile values for 12 dichotomous traits. Prevalence (a) was calculated as the number of cases in total individuals above the percentile threshold of PRS and odds ratios (b) were calculated by comparing those with a high polygenic score above the percentile threshold against the whole UKB population. This is the extended figure of Figure 6 for additional values above 90% percentile or the 9^th^ decile. The dot points represent the mean values and the error-bars denote the 95% confidence intervals of the mean values averaged over 12 dichotomous traits.

***
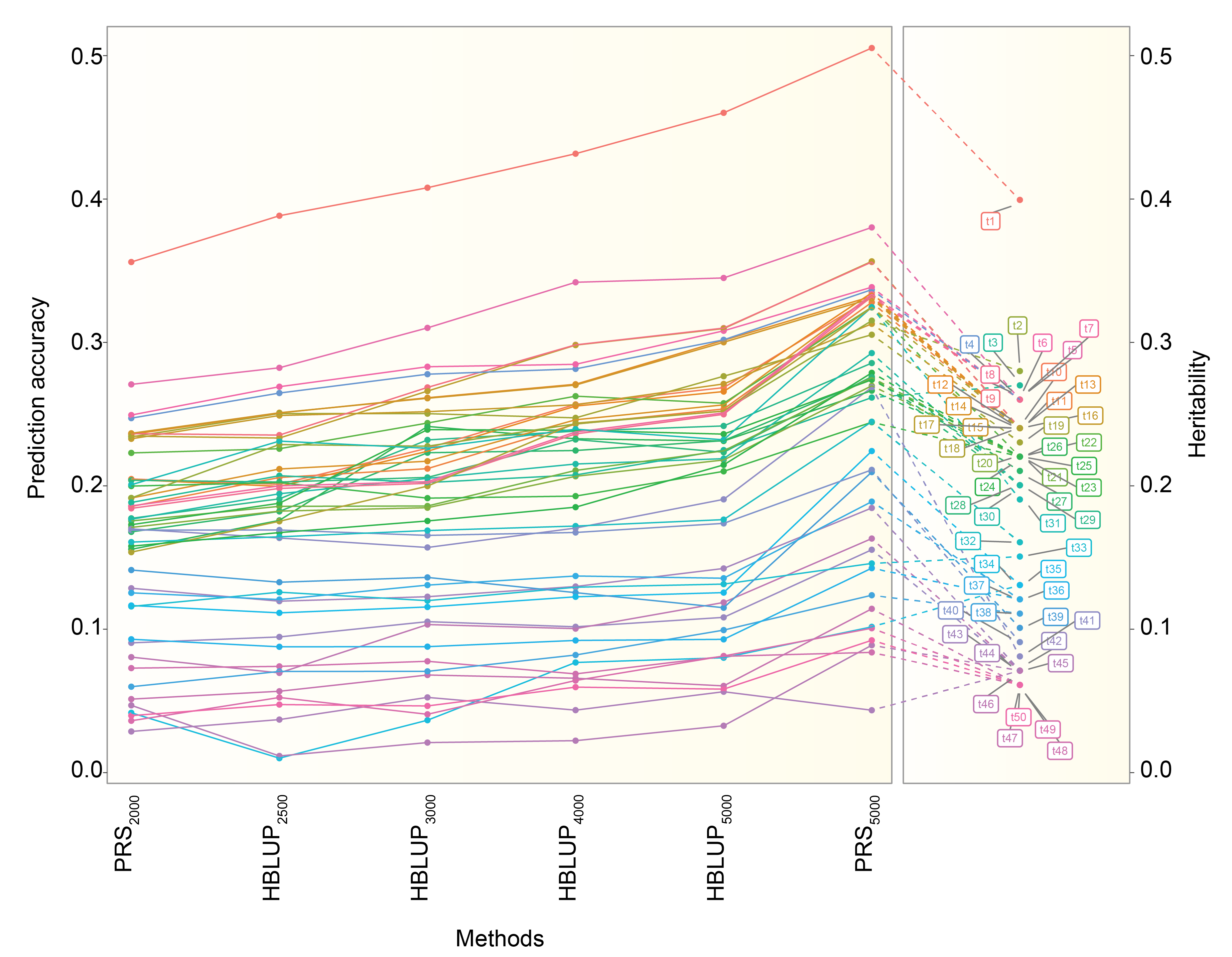
***

**Supplementary Figure 7. Sensitivity analysis for polygenic prediction accuracy with different numbers of ungenotyped relatives and narrow-sense heritability for 50 traits.** Here we assessed prediction performance in the small-scale design considering 500, 1000, 2000 and 3000 ungenotyped relatives added to discovery sample and PRS with reduced number of genotyped individuals and with all individuals genotyped. ‘t’ stands for each trait in 50 traits that can be found in Supplementary Table 8. Left panel demonstrates prediction accuracy for different number of ungenotyped individuals. Right panel show heritability for each trait in 50 traits. The dashed lines connect each trait with its narrow-sense heritability obtained from Neale et al. The subscript in the name of each method represents the number of individuals in the discovery sample. PRS_2000_: Polygenic Risk Score with 2000 genotyped individuals after removing 3000 ungenotyped relatives; HBLUP_2500_, HBLUP_3000_, HBLUP_4000_, HBLUP_5000_: Best Linear Unbiased Prediction with genomic-pedigree relationship **H**-matrix including 2000 genotyped and 500, 1000, 2000, 3000 ungenotyped individuals in discovery sample, respectively.


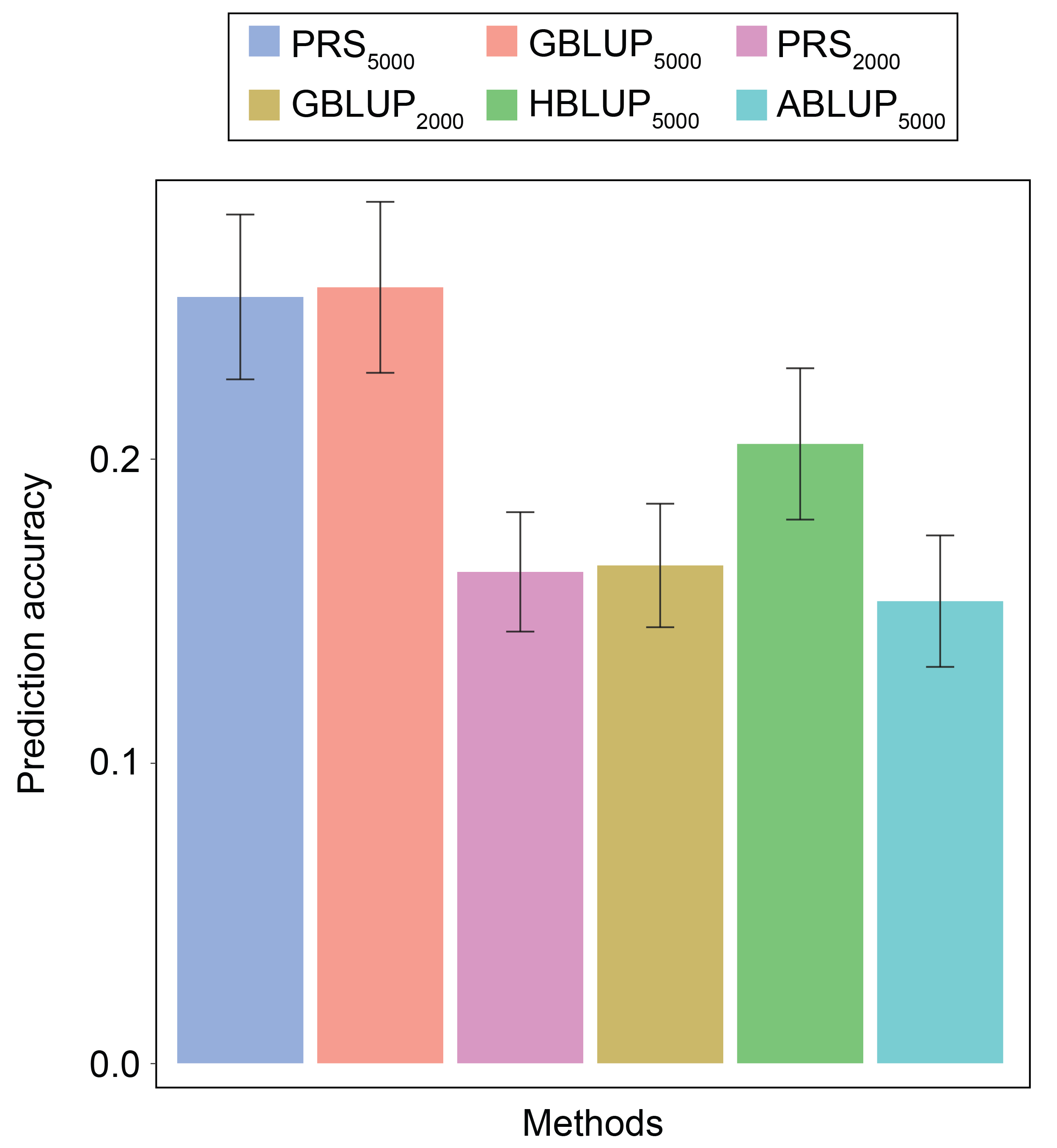


**Supplementary Figure 8. Prediction accuracy for genotyped and ungenotyped designs.** The subscript in the name of each method represents the number of individuals in the discovery sample. PRS_5000_: PRS with 5000 individuals in the discovery sample, PRS_2000_: Polygenic Risk Score with 2000 genotyped individuals after removing 3000 ungenotyped relatives; HBLUP_5000_^,^ ABLUP_5000_: Best Linear Unbiased Prediction including 2000 genotyped and 3000 ungenotyped individuals in discovery sample with genomic-pedigree relationship **H**-matrix and **A**-matrix, respectively. The main bars represent the mean values averaged over the analyses of 50 traits. The error-bars show the 95% confidence intervals of the mean values.

**SUPPLEMENTARY TABLES**

**Supplementary Table 1. Detailed information for 50 traits.**

| Trait name | Trait type | Type of values | Heritability | | | | | |
| --- | --- | --- | --- | --- | --- | --- | --- | --- |
|  |  |  | Small-scale design | | | | Large-scale design | Neale et al. |
|  |  |  | Unrelated | 3^rd^ degree | 2^nd^ degree | 1^st^ degree | Unrelated |  |
| Qualifications: None of the above | Lifestyle | Binary | 0.082 | 0.3512 | 0.2405 | 0.1749 | 0.0913 | 0.095 |
| Past tobacco smoking | Lifestyle | Continuous | 0.0751 | 0.2422 | 0.2127 | 0.2594 | 0.0877 | 0.089 |
| Alcohol intake frequency. | Lifestyle | Continuous | 0.1191 | 0.2342 | 0.2815 | 0.3576 | 0.0771 | 0.081 |
| Ever smoked | Lifestyle | Binary | 0.1038 | 0.176 | 0.1348 | 0.2377 | 0.0776 | 0.074 |
| Qualifications: College or University degree | Lifestyle | Binary | 0.3157 | 0.3742 | 0.4182 | 0.4004 | 0.1497 | 0.161 |
| Sensitivity / hurt feelings | Mood | Binary | 0.0957 | 0.1189 | 0.1333 | 0.1741 | 0.0602 | 0.062 |
| Mood swings | Mood | Binary | 0.065 | 0.1906 | 0.105 | 0.2069 | 0.0725 | 0.07 |
| Miserableness | Mood | Binary | 0.0138 | 0.1499 | 0.1338 | 0.1743 | 0.0639 | 0.063 |
| Irritability | Mood | Binary | 0.1483 | 0.1199 | 0.0485 | 0.1138 | 0.0667 | 0.066 |
| Fed-up feelings | Mood | Binary | 0.1472 | 0.1819 | 0.0895 | 0.1499 | 0.0714 | 0.071 |
| Nervous feelings | Mood | Binary | 0.1116 | 0.1341 | 0.1367 | 0.146 | 0.0682 | 0.064 |
| Worry too long after embarrassment | Mood | Binary | 0.0331 | 0.0528 | 0.1055 | 0.148 | 0.0645 | 0.064 |
| Neuroticism score | Mood | Continuous | 0.0286 | 0.2282 | 0.1967 | 0.2187 | 0.1177 | 0.115 |
| Leg predicted mass (left) | Physical | Continuous | 0.1711 | 0.3039 | 0.2792 | 0.2626 | 0.244 | 0.242 |
| Arm fat-free mass (right) | Physical | Continuous | 0.0634 | 0.2582 | 0.2415 | 0.2341 | 0.2436 | 0.242 |
| Arm fat-free mass (left) | Physical | Continuous | 0.102 | 0.2606 | 0.2556 | 0.2522 | 0.2342 | 0.241 |
| Arm predicted mass (right) | Physical | Continuous | 0.1083 | 0.2485 | 0.2386 | 0.2319 | 0.2374 | 0.241 |
| Leg fat-free mass (right) | Physical | Continuous | 0.0924 | 0.305 | 0.294 | 0.2694 | 0.2462 | 0.241 |
| Arm predicted mass (left) | Physical | Continuous | 0.1057 | 0.2588 | 0.2555 | 0.2542 | 0.2383 | 0.24 |
| Leg predicted mass (right) | Physical | Continuous | 0.0885 | 0.3011 | 0.2872 | 0.2643 | 0.2462 | 0.24 |
| Impedance of whole body | Physical | Continuous | 0.2748 | 0.3489 | 0.3201 | 0.3537 | 0.2427 | 0.24 |
| Impedance of leg (left) | Physical | Continuous | 0.3924 | 0.3643 | 0.4231 | 0.4188 | 0.2285 | 0.225 |
| Impedance of leg (right) | Physical | Continuous | 0.2512 | 0.3491 | 0.4091 | 0.4347 | 0.2236 | 0.223 |
| Impedance of arm (right) | Physical | Continuous | 0.1994 | 0.2702 | 0.256 | 0.2808 | 0.2081 | 0.22 |
| Whole body water mass | Physical | Continuous | 0.0345 | 0.2863 | 0.2743 | 0.2718 | 0.2714 | 0.261 |
| Whole body fat-free mass | Physical | Continuous | 0.0776 | 0.2862 | 0.2731 | 0.2696 | 0.2723 | 0.261 |
| Basal metabolic rate | Physical | Continuous | 0.0884 | 0.314 | 0.3047 | 0.3074 | 0.2596 | 0.258 |
| Trunk fat-free mass | Physical | Continuous | 0.0573 | 0.2784 | 0.2626 | 0.2732 | 0.2693 | 0.258 |
| Trunk predicted mass | Physical | Continuous | 0.1034 | 0.2731 | 0.256 | 0.2663 | 0.2707 | 0.256 |
| Leg fat-free mass (left) | Physical | Continuous | 0.0573 | 0.3081 | 0.2861 | 0.2677 | 0.2437 | 0.242 |
| Blood clot, DVT, bronchitis, emphysema,  asthma, rhinitis, eczema, allergy diagnosed by  doctor: Hayfever, allergic rhinitis or eczema | Physical | Binary | -0.0915 | 0.0985 | 0.1383 | 0.1873 | 0.066 | 0.067 |
| Vascular/heart problems diagnosed by doctor: High blood pressure | Physical | Binary | 0.2747 | 0.2365 | 0.1573 | 0.1345 | 0.1178 | 0.116 |
| Arm fat percentage (left) | Physical | Continuous | 0.1307 | 0.2373 | 0.2245 | 0.3255 | 0.2121 | 0.206 |
| Leg fat mass (right) | Physical | Continuous | 0.095 | 0.2716 | 0.2162 | 0.4072 | 0.1894 | 0.215 |
| Whole body fat mass | Physical | Continuous | 0.1902 | 0.3954 | 0.3498 | 0.489 | 0.2233 | 0.218 |
| Leg fat mass (left) | Physical | Continuous | 0.0905 | 0.2623 | 0.2101 | 0.4012 | 0.1879 | 0.216 |
| Arm fat mass (right) | Physical | Continuous | 0.2241 | 0.3054 | 0.1171 | 0.4746 | 0.1261 | 0.216 |
| Body fat percentage | Physical | Continuous | 0.0843 | 0.2623 | 0.2414 | 0.3074 | 0.2119 | 0.203 |
| Arm fat percentage (right) | Physical | Continuous | 0.1476 | 0.236 | 0.2237 | 0.3215 | 0.2047 | 0.202 |
| Pulse rate, automated reading | Physical | Continuous | 0.1546 | 0.1176 | 0.2312 | 0.2311 | 0.1413 | 0.146 |
| Comparative body size at age 10 | Physical | Continuous | 0.1227 | 0.2135 | 0.1618 | 0.1694 | 0.1384 | 0.135 |
| Sitting height | Physical | Continuous | 0.2173 | 0.3711 | 0.3819 | 0.3901 | 0.3007 | 0.281 |
| Body mass index (BMI) | Physical | Continuous | 0.2636 | 0.4245 | 0.3931 | 0.5037 | 0.2303 | 0.231 |
| Weight | Physical | Continuous | 0.2874 | 0.4352 | 0.4214 | 0.4929 | 0.2454 | 0.243 |
| Diastolic blood pressure, automated reading | Physical | Continuous | 0.0105 | 0.2875 | 0.232 | 0.281 | 0.1314 | 0.131 |
| Systolic blood pressure, automated reading | Physical | Continuous | 0.0861 | 0.3109 | 0.1543 | 0.1594 | 0.1423 | 0.123 |
| Waist circumference | Physical | Continuous | 0.2117 | 0.3193 | 0.3384 | 0.3761 | 0.1957 | 0.189 |
| Hip circumference | Physical | Continuous | 0.1677 | 0.3786 | 0.343 | 0.4699 | 0.2027 | 0.201 |
| Standing height | Physical | Continuous | 0.3941 | 0.4254 | 0.4835 | 0.5133 | 0.4258 | 0.401 |
| Heel bone mineral density (BMD) T-score, automated | Physical | Continuous | 0.3561 | 0.3973 | 0.3613 | 0.4707 | 0.2386 | 0.27 |

**Supplementary Table 2. The average proportion of relatives used in the large-scale design for each level of kinship coefficients.**

|  | 1^st^ degree  (parent-offspring  and full sibs) | 2^nd^ degree | 3^rd^ degree |
| --- | --- | --- | --- |
| Kinship coefficient | ($\frac{1}{2^{5/2}}, \frac{1}{2^{3/2}})$ | ($\frac{1}{2^{7/2}}, \frac{1}{2^{5/2}})$ | ($\frac{1}{2^{9/2}}, \frac{1}{2^{7/2}})$ |
| Proportion of relatives  in the large-scale design  (average [minimum – maximum value]) | 14.3%  [12.3 - 14.6%] | 5.3%  [4.5 - 5.4%] | 23.9%  [19.9 - 24.4%] |
| Number of relatives  with phenotype information  in the large-scale design (average [minimum – maximum value]) | 40,023  [20,389 - 42,295] | 14,789 [7,431 - 15,716] | 66,862 [32,839 - 70,518] |
| Proportion of relatives  in the small-scale design  (average [minimum – maximum value]) | 100% [100% - 100%] | 100% [100% - 100%] | 100% [100% - 100%] |
| Number of relatives  with phenotype information  in the small-scale design (average [minimum – maximum value]) | 6000 [6000 – 6000] | 6000 [6000 – 6000] | 6000 [6000 – 6000] |
| ‘Proportion of relatives’ in the large/small-scale design was calculated by the fraction of number of close relatives over the total number of samples in a large/small-scale design for each trait. | | | |

**Supplementary Table 3. Number of individuals for unrelated sample and close relatives.**

| Trait name | Number of  individuals in  unrelated sample | | Number of  individuals in 3^rd^ degree relatives | | Number of  individuals in 2^nd^ degree relatives | | Number of  individuals in  1^st^ degree relatives | | Proportion of  substituted sample (%) | | |
| --- | --- | --- | --- | --- | --- | --- | --- | --- | --- | --- | --- |
|  | Discovery | Target | Discovery | Target | Discovery | Target | Discovery | Target | 3^rd^ degree | 2^nd^ degree | 1^st^ degree |
| Leg predicted mass (left) | 227018 | 56754 | 34253 | 34238 | 7550 | 7602 | 20544 | 20504 | 24.14 | 5.34 | 14.47 |
| Arm fat-free mass (right) | 226992 | 56748 | 34246 | 34232 | 7549 | 7601 | 20541 | 20501 | 24.13 | 5.34 | 14.46 |
| Arm fat-free mass (left) | 226951 | 56738 | 34233 | 34220 | 7545 | 7597 | 20533 | 20493 | 24.13 | 5.34 | 14.46 |
| Arm predicted mass (right) | 226990 | 56747 | 34245 | 34231 | 7548 | 7600 | 20541 | 20501 | 24.13 | 5.34 | 14.46 |
| Leg fat-free mass (right) | 227038 | 56759 | 34258 | 34242 | 7551 | 7604 | 20548 | 20507 | 24.14 | 5.34 | 14.47 |
| Arm predicted mass (left) | 226942 | 56736 | 34232 | 34219 | 7545 | 7597 | 20530 | 20490 | 24.13 | 5.34 | 14.46 |
| Leg predicted mass (right) | 227038 | 56759 | 34257 | 34241 | 7550 | 7603 | 20548 | 20507 | 24.14 | 5.34 | 14.47 |
| Impedance of whole body | 227039 | 56760 | 34258 | 34241 | 7551 | 7604 | 20550 | 20509 | 24.14 | 5.34 | 14.47 |
| Impedance of leg (left) | 227047 | 56762 | 34258 | 34241 | 7551 | 7604 | 20552 | 20511 | 24.14 | 5.34 | 14.47 |
| Impedance of leg (right) | 227051 | 56763 | 34258 | 34241 | 7551 | 7604 | 20552 | 20511 | 24.14 | 5.34 | 14.47 |
| Impedance of arm (right) | 227035 | 56759 | 34257 | 34242 | 7548 | 7601 | 20548 | 20506 | 24.14 | 5.34 | 14.47 |
| Whole body water mass | 227060 | 56765 | 34262 | 34245 | 7552 | 7605 | 20555 | 20514 | 24.14 | 5.34 | 14.47 |
| Whole body fat-free mass | 227040 | 56760 | 34254 | 34237 | 7550 | 7603 | 20553 | 20512 | 24.13 | 5.34 | 14.47 |
| Basal metabolic rate | 227056 | 56764 | 34261 | 34244 | 7551 | 7604 | 20553 | 20512 | 24.14 | 5.34 | 14.47 |
| Trunk fat-free mass | 226861 | 56715 | 34213 | 34203 | 7539 | 7591 | 20520 | 20481 | 24.13 | 5.34 | 14.46 |
| Trunk predicted mass | 226835 | 56709 | 34210 | 34201 | 7538 | 7590 | 20517 | 20478 | 24.13 | 5.34 | 14.46 |
| Leg fat-free mass (left) | 227020 | 56755 | 34253 | 34238 | 7551 | 7604 | 20544 | 20504 | 24.14 | 5.34 | 14.46 |
| Qualifications: None of the above | 228982 | 57245 | 34729 | 34707 | 7687 | 7727 | 20839 | 20797 | 24.26 | 5.39 | 14.55 |
| Blood clot, DVT, bronchitis, emphysema,  asthma, rhinitis, eczema, allergy diagnosed by  doctor: Hayfever, allergic rhinitis or eczema | 230785 | 57696 | 35185 | 35183 | 7800 | 7857 | 21126 | 21084 | 24.39 | 5.43 | 14.63 |
| Sensitivity / hurt feelings | 224657 | 56164 | 33649 | 33641 | 7453 | 7510 | 20058 | 20014 | 23.96 | 5.33 | 14.27 |
| Vascular/heart problems diagnosed by doctor:  High blood pressure | 230711 | 57678 | 35168 | 35171 | 7801 | 7859 | 21110 | 21067 | 24.39 | 5.43 | 14.63 |
| Arm fat percentage (left) | 226854 | 56714 | 34215 | 34204 | 7544 | 7595 | 20514 | 20475 | 24.13 | 5.34 | 14.45 |
| Leg fat mass (right) | 226914 | 56729 | 34230 | 34216 | 7549 | 7601 | 20524 | 20484 | 24.13 | 5.34 | 14.46 |
| Whole body fat mass | 226542 | 56636 | 34168 | 34151 | 7539 | 7591 | 20459 | 20421 | 24.13 | 5.34 | 14.44 |
| Leg fat mass (left) | 226910 | 56728 | 34229 | 34216 | 7548 | 7600 | 20521 | 20482 | 24.13 | 5.34 | 14.46 |
| Arm fat mass (right) | 226874 | 56719 | 34223 | 34210 | 7546 | 7597 | 20517 | 20478 | 24.13 | 5.34 | 14.46 |
| Body fat percentage | 226738 | 56684 | 34202 | 34186 | 7544 | 7598 | 20491 | 20452 | 24.13 | 5.34 | 14.45 |
| Arm fat percentage (right) | 226892 | 56723 | 34225 | 34213 | 7548 | 7599 | 20520 | 20481 | 24.13 | 5.34 | 14.46 |
| Pulse rate, automated reading | 215621 | 53905 | 31639 | 31638 | 6983 | 7037 | 18935 | 18892 | 23.48 | 5.2 | 14.03 |
| Past tobacco smoking | 213301 | 53325 | 30645 | 30721 | 6528 | 6587 | 18243 | 18224 | 23.02 | 4.92 | 13.68 |
| Alcohol intake frequency. | 231068 | 57767 | 35262 | 35256 | 7830 | 7886 | 21168 | 21127 | 24.41 | 5.44 | 14.64 |
| Comparative body size at age 10 | 227319 | 56830 | 34307 | 34307 | 7582 | 7647 | 20560 | 20526 | 24.15 | 5.36 | 14.46 |
| Mood swings | 225826 | 56456 | 33931 | 33925 | 7469 | 7509 | 20281 | 20245 | 24.04 | 5.31 | 14.36 |
| Miserableness | 227405 | 56851 | 34382 | 34365 | 7594 | 7647 | 20558 | 20523 | 24.18 | 5.36 | 14.45 |
| Irritability | 221109 | 55277 | 32828 | 32778 | 7247 | 7278 | 19522 | 19491 | 23.74 | 5.26 | 14.12 |
| Fed-up feelings | 226550 | 56638 | 34132 | 34101 | 7513 | 7571 | 20392 | 20350 | 24.09 | 5.33 | 14.39 |
| Nervous feelings | 225328 | 56332 | 33822 | 33834 | 7494 | 7544 | 20180 | 20134 | 24.02 | 5.34 | 14.31 |
| Worry too long after embarrassment | 221890 | 55472 | 33060 | 33024 | 7241 | 7315 | 19629 | 19624 | 23.83 | 5.25 | 14.15 |
| Neuroticism score | 188086 | 47022 | 24940 | 24914 | 5277 | 5299 | 14472 | 14453 | 21.2 | 4.5 | 12.3 |
| Sitting height | 230351 | 57588 | 35085 | 35080 | 7780 | 7835 | 21072 | 21031 | 24.37 | 5.42 | 14.62 |
| Ever smoked | 209918 | 52480 | 29781 | 29792 | 6445 | 6474 | 17781 | 17748 | 22.7 | 4.92 | 13.54 |
| Body mass index (BMI) | 230337 | 57584 | 35081 | 35076 | 7765 | 7823 | 21066 | 21026 | 24.37 | 5.41 | 14.62 |
| Weight | 230424 | 57606 | 35103 | 35099 | 7768 | 7827 | 21082 | 21042 | 24.37 | 5.41 | 14.62 |
| Diastolic blood pressure, automated reading | 210633 | 52658 | 30389 | 30408 | 6641 | 6680 | 18154 | 18112 | 23.09 | 5.06 | 13.77 |
| Systolic blood pressure, automated reading | 210629 | 52657 | 30388 | 30407 | 6641 | 6680 | 18154 | 18112 | 23.09 | 5.06 | 13.77 |
| Waist circumference | 230684 | 57671 | 35157 | 35156 | 7800 | 7856 | 21117 | 21076 | 24.38 | 5.43 | 14.63 |
| Hip circumference | 230653 | 57663 | 35151 | 35149 | 7798 | 7854 | 21116 | 21075 | 24.38 | 5.43 | 14.63 |
| Standing height | 230569 | 57642 | 35122 | 35124 | 7791 | 7847 | 21101 | 21060 | 24.37 | 5.43 | 14.63 |
| Qualifications: College or University degree | 228982 | 57245 | 34729 | 34707 | 7687 | 7727 | 20839 | 20797 | 24.26 | 5.39 | 14.55 |
| Heel bone mineral density (BMD) T-score, automated | 132228 | 33057 | 16422 | 16417 | 3709 | 3722 | 10205 | 10184 | 19.87 | 4.5 | 12.34 |
| ‘Proportion of substituted sample' was calculated by the fraction of number of close relatives over the total number of samples in a large-scale design for each trait. | | | | | | | | | | | |

**Supplementary Table 4. Empirical prediction accuracy for 50 traits in large-scale and small-scale designs with varying reference sample sizes.**

| Trait name | Large-scale design | | | | Small-scale design | | | | | |
| --- | --- | --- | --- | --- | --- | --- | --- | --- | --- | --- |
|  | Ref. size ~ 223,215 | | | | Ref. size = 5,000 | | | | Ref. size = 10,000 | Ref. size = 15,000 |
|  | Unrelated Sample | 3rd degree Sample | 2nd degree Sample | 1st degree Sample | Unrelated Sample | 3rd degree Sample | 2nd degree Sample | 1st degree Sample | 1st degree Sample | 1st degree Sample |
| Leg predicted mass (left) | 0.296 | 0.3 | 0.297 | 0.312 | 0.101 | 0.203 | 0.251 | 0.356 | 0.368 | 0.376 |
| Arm fat-free mass (right) | 0.301 | 0.304 | 0.291 | 0.318 | 0.098 | 0.193 | 0.249 | 0.331 | 0.368 | 0.372 |
| Arm fat-free mass (left) | 0.303 | 0.302 | 0.301 | 0.314 | 0.068 | 0.191 | 0.241 | 0.332 | 0.369 | 0.375 |
| Arm predicted mass (right) | 0.306 | 0.302 | 0.296 | 0.32 | 0.095 | 0.193 | 0.252 | 0.335 | 0.369 | 0.375 |
| Leg fat-free mass (right) | 0.304 | 0.303 | 0.303 | 0.315 | 0.096 | 0.205 | 0.266 | 0.335 | 0.381 | 0.385 |
| Arm predicted mass (left) | 0.303 | 0.304 | 0.303 | 0.318 | 0.109 | 0.18 | 0.239 | 0.337 | 0.373 | 0.377 |
| Leg predicted mass (right) | 0.304 | 0.303 | 0.303 | 0.316 | 0.096 | 0.203 | 0.264 | 0.334 | 0.382 | 0.387 |
| Impedance of whole body | 0.289 | 0.284 | 0.289 | 0.3 | 0.063 | 0.136 | 0.187 | 0.314 | 0.327 | 0.33 |
| Impedance of leg (left) | 0.272 | 0.266 | 0.272 | 0.282 | 0.158 | 0.111 | 0.211 | 0.272 | 0.331 | 0.329 |
| Impedance of leg (right) | 0.277 | 0.273 | 0.277 | 0.291 | 0.07 | 0.118 | 0.192 | 0.278 | 0.347 | 0.344 |
| Impedance of arm (right) | 0.263 | 0.256 | 0.265 | 0.275 | 0.053 | 0.116 | 0.168 | 0.322 | 0.333 | 0.338 |
| Whole body water mass | 0.329 | 0.323 | 0.329 | 0.34 | 0.091 | 0.211 | 0.27 | 0.342 | 0.396 | 0.4 |
| Whole body fat-free mass | 0.333 | 0.33 | 0.331 | 0.345 | 0.053 | 0.212 | 0.271 | 0.381 | 0.402 | 0.406 |
| Basal metabolic rate | 0.323 | 0.323 | 0.324 | 0.337 | 0.076 | 0.206 | 0.269 | 0.343 | 0.389 | 0.395 |
| Trunk fat-free mass | 0.33 | 0.33 | 0.331 | 0.345 | 0.042 | 0.197 | 0.253 | 0.332 | 0.389 | 0.392 |
| Trunk predicted mass | 0.328 | 0.329 | 0.326 | 0.344 | 0.134 | 0.221 | 0.26 | 0.33 | 0.387 | 0.391 |
| Leg fat-free mass (left) | 0.298 | 0.301 | 0.295 | 0.309 | 0.085 | 0.202 | 0.273 | 0.357 | 0.37 | 0.377 |
| Qualifications: None of the above | 0.158 | 0.165 | 0.159 | 0.171 | 0.014 | 0.182 | 0.135 | 0.228 | 0.153 | 0.157 |
| Blood clot, DVT, bronchitis, emphysema,  asthma, rhinitis, eczema, allergy diagnosed by  doctor: Hayfever, allergic rhinitis or eczema | 0.126 | 0.125 | 0.126 | 0.133 | 0.032 | 0.121 | 0.076 | 0.158 | 0.14 | 0.143 |
| Sensitivity / hurt feelings | 0.098 | 0.1 | 0.098 | 0.1 | 0.072 | 0.036 | 0.072 | 0.123 | 0.117 | 0.089 |
| Vascular/heart problems diagnosed by doctor:  High blood pressure | 0.177 | 0.182 | 0.176 | 0.18 | 0.061 | 0.13 | 0.076 | 0.151 | 0.13 | 0.15 |
| Arm fat percentage (left) | 0.259 | 0.264 | 0.261 | 0.27 | 0.048 | 0.143 | 0.162 | 0.277 | 0.322 | 0.337 |
| Leg fat mass (right) | 0.239 | 0.255 | 0.242 | 0.256 | 0.076 | 0.109 | 0.094 | 0.245 | 0.304 | 0.305 |
| Whole body fat mass | 0.273 | 0.278 | 0.276 | 0.288 | 0.079 | 0.143 | 0.099 | 0.285 | 0.342 | 0.356 |
| Leg fat mass (left) | 0.238 | 0.246 | 0.239 | 0.248 | 0.057 | 0.099 | 0.105 | 0.276 | 0.305 | 0.308 |
| Arm fat mass (right) | 0.188 | 0.194 | 0.19 | 0.189 | 0.076 | 0.041 | 0.037 | 0.271 | 0.281 | 0.27 |
| Body fat percentage | 0.278 | 0.282 | 0.279 | 0.287 | 0.091 | 0.114 | 0.093 | 0.275 | 0.329 | 0.347 |
| Arm fat percentage (right) | 0.257 | 0.261 | 0.258 | 0.27 | 0.063 | 0.137 | 0.145 | 0.289 | 0.327 | 0.347 |
| Pulse rate, automated reading | 0.197 | 0.187 | 0.199 | 0.202 | 0.047 | 0.032 | 0.11 | 0.107 | 0.107 | 0.114 |
| Past tobacco smoking | 0.148 | 0.142 | 0.147 | 0.154 | 0.026 | 0.068 | 0.096 | 0.217 | 0.161 | 0.146 |
| Alcohol intake frequency. | 0.13 | 0.135 | 0.133 | 0.145 | 0.042 | 0.109 | 0.139 | 0.26 | 0.299 | 0.296 |
| Comparative body size at age 10 | 0.197 | 0.194 | 0.197 | 0.203 | 0.071 | 0.062 | 0.058 | 0.117 | 0.119 | 0.117 |
| Mood swings | 0.103 | 0.11 | 0.105 | 0.104 | 0.029 | 0.112 | 0.078 | 0.192 | 0.192 | 0.181 |
| Miserableness | 0.103 | 0.108 | 0.102 | 0.106 | 0.016 | 0.039 | 0.119 | 0.09 | 0.119 | 0.113 |
| Irritability | 0.097 | 0.096 | 0.099 | 0.096 | 0.043 | 0.017 | 0.015 | 0.049 | 0.075 | 0.073 |
| Fed-up feelings | 0.109 | 0.118 | 0.109 | 0.117 | 0.043 | 0.094 | 0.013 | 0.09 | 0.11 | 0.109 |
| Nervous feelings | 0.101 | 0.092 | 0.1 | 0.105 | 0.049 | 0.059 | 0.07 | 0.108 | 0.08 | 0.073 |
| Worry too long after embarrassment | 0.108 | 0.108 | 0.106 | 0.107 | 0.035 | 0.029 | 0.019 | 0.097 | 0.079 | 0.084 |
| Neuroticism score | 0.156 | 0.156 | 0.156 | 0.155 | 0.033 | 0.102 | 0.091 | 0.128 | 0.123 | 0.143 |
| Sitting height | 0.353 | 0.36 | 0.353 | 0.361 | 0.097 | 0.22 | 0.212 | 0.325 | 0.431 | 0.42 |
| Ever smoked | 0.114 | 0.11 | 0.114 | 0.123 | 0.014 | 0.112 | 0.078 | 0.171 | 0.132 | 0.12 |
| Body mass index (BMI) | 0.263 | 0.266 | 0.267 | 0.277 | 0.053 | 0.184 | 0.175 | 0.306 | 0.319 | 0.335 |
| Weight | 0.299 | 0.299 | 0.3 | 0.311 | 0.122 | 0.223 | 0.166 | 0.328 | 0.359 | 0.376 |
| Diastolic blood pressure, automated reading | 0.194 | 0.195 | 0.19 | 0.198 | 0.031 | 0.183 | 0.143 | 0.229 | 0.203 | 0.202 |
| Systolic blood pressure, automated reading | 0.188 | 0.19 | 0.187 | 0.194 | 0.052 | 0.148 | 0.133 | 0.193 | 0.23 | 0.245 |
| Waist circumference | 0.255 | 0.257 | 0.255 | 0.268 | 0.068 | 0.15 | 0.173 | 0.327 | 0.313 | 0.326 |
| Hip circumference | 0.27 | 0.263 | 0.268 | 0.281 | 0.017 | 0.145 | 0.168 | 0.296 | 0.322 | 0.34 |
| Standing height | 0.45 | 0.447 | 0.451 | 0.463 | 0.182 | 0.322 | 0.361 | 0.505 | 0.586 | 0.572 |
| Qualifications: College or University degree | 0.215 | 0.22 | 0.213 | 0.232 | 0.097 | 0.178 | 0.166 | 0.249 | 0.293 | 0.292 |
| Heel bone mineral density (BMD) T-score, automated | 0.241 | 0.245 | 0.239 | 0.255 | 0.148 | 0.176 | 0.097 | 0.269 | 0.291 | 0.298 |
|  |  |  |  |  |  |  |  |  |  |  |
| **Average** | 0.235 | 0.236 | 0.235 | 0.245 | 0.069 | 0.142 | 0.158 | 0.257 | 0.279 | 0.283 |

Ref. size: reference sample size.

**Supplementary Table 5. The effective number of chromosome segments for 50 traits in the experiments with unrelated, 3^rd^, 2^nd^, and 1^st^ degree relatives in the small-scale design.**

| Trait name | Unrelated Sample | 3^rd^ degree Sample | 2^nd^ degree Sample | 1^st^ degree Sample |
| --- | --- | --- | --- | --- |
| Leg predicted mass (left) | 50899.72 | 23866.79 | 22536.35 | 10621.47 |
| Arm fat-free mass (right) | 50805.44 | 23866.79 | 22536.35 | 10621.47 |
| Arm fat-free mass (left) | 50790.87 | 23866.79 | 22536.35 | 10713.95 |
| Arm predicted mass (right) | 50858.68 | 23866.79 | 22536.35 | 10621.47 |
| Leg fat-free mass (right) | 50865.35 | 23866.79 | 22478.64 | 10658.75 |
| Arm predicted mass (left) | 50707.47 | 23866.79 | 22536.35 | 10713.95 |
| Leg predicted mass (right) | 50865.35 | 23866.79 | 22478.64 | 10658.75 |
| Impedance of whole body | 50755.64 | 23866.79 | 22478.64 | 10658.75 |
| Impedance of leg (left) | 50855.52 | 23866.79 | 22478.64 | 10658.75 |
| Impedance of leg (right) | 50756.67 | 23866.79 | 22478.64 | 10658.75 |
| Impedance of arm (right) | 50762.84 | 23866.79 | 22478.64 | 10668.44 |
| Whole body water mass | 50840.12 | 23866.79 | 22491.76 | 10658.75 |
| Whole body fat-free mass | 50730.28 | 23866.79 | 22491.76 | 10287.54 |
| Basal metabolic rate | 50863.21 | 23866.79 | 22478.64 | 10658.75 |
| Trunk fat-free mass | 50856.85 | 24128.81 | 22536.35 | 10670.7 |
| Trunk predicted mass | 50815.41 | 24078.37 | 22538.79 | 10670.7 |
| Leg fat-free mass (left) | 50854.82 | 23866.79 | 22414.35 | 10621.47 |
| Qualifications: None of the above | 50894.44 | 23370.92 | 22620.7 | 10731.83 |
| Blood clot, DVT, bronchitis, emphysema,  asthma, rhinitis, eczema, allergy diagnosed by  doctor: Hayfever, allergic rhinitis or eczema | 50788.87 | 24310.07 | 21952.03 | 10460.52 |
| Sensitivity / hurt feelings | 50845.92 | 23845.91 | 23061.86 | 10637.39 |
| Vascular/heart problems diagnosed by doctor:  High blood pressure | 50732.43 | 23767.44 | 21987.46 | 10604.3 |
| Arm fat percentage (left) | 50827.74 | 23866.79 | 22845.4 | 10205.48 |
| Leg fat mass (right) | 50763.96 | 23866.79 | 22952.27 | 10342.47 |
| Whole body fat mass | 50917.18 | 24104.8 | 23179.58 | 10273.03 |
| Leg fat mass (left) | 50855.03 | 23866.79 | 22952.27 | 10205.48 |
| Arm fat mass (right) | 50862.16 | 23866.79 | 22845.4 | 10205.48 |
| Body fat percentage | 50758.8 | 24676.93 | 22643.75 | 10193.98 |
| Arm fat percentage (right) | 50811.77 | 23866.79 | 22845.4 | 10205.48 |
| Pulse rate, automated reading | 50859.84 | 24102.61 | 22715.4 | 10669.26 |
| Past tobacco smoking | 50797.38 | 23975.48 | 23945.99 | 10684.39 |
| Alcohol intake frequency. | 50848.33 | 24202.01 | 21925.67 | 10556.97 |
| Comparative body size at age 10 | 50813.36 | 23838.22 | 22467.85 | 10419.09 |
| Mood swings | 50851.36 | 24234.91 | 22638.49 | 10583.29 |
| Miserableness | 50837.62 | 24522.61 | 22607.35 | 10235.63 |
| Irritability | 50885.22 | 23281.1 | 23160.12 | 10408.95 |
| Fed-up feelings | 50827.64 | 24041.51 | 22101.9 | 10682.12 |
| Nervous feelings | 50754.81 | 22561.61 | 22380.23 | 10572.01 |
| Worry too long after embarrassment | 50853.61 | 24286.09 | 22801.02 | 10412.29 |
| Neuroticism score | 50822.98 | 25554.2 | 26318.65 | 12262.88 |
| Sitting height | 50879.29 | 23996.04 | 22054.62 | 10501.44 |
| Ever smoked | 50719.75 | 24027.02 | 23964.61 | 11311.73 |
| Body mass index (BMI) | 50734.22 | 24351.88 | 21992.62 | 10581.76 |
| Weight | 50749.91 | 23618.01 | 22202.29 | 10564.54 |
| Diastolic blood pressure, automated reading | 50839.47 | 24528.47 | 23549.52 | 10684.25 |
| Systolic blood pressure, automated reading | 50867.08 | 24528.47 | 23549.52 | 10897.82 |
| Waist circumference | 50811.82 | 24775.63 | 22038.42 | 10673.41 |
| Hip circumference | 50778.9 | 23735.59 | 21991.55 | 10483.4 |
| Standing height | 50861.08 | 24108.8 | 22260.01 | 10688.47 |
| Qualifications: College or University degree | 50894.44 | 23370.92 | 22620.7 | 10731.83 |
| Heel bone mineral density (BMD) T-score, automated | 50722.62 | 24854.68 | 27108.52 | 12478.02 |

**Supplementary Table 6. Theoretical prediction accuracy with both narrow-sense and family-based heritability in the small-scale design.**

| Trait name | Theoretical prediction accuracy with  Narrow-sense heritability | | | | Theoretical prediction accuracy with  Family-based heritability | | | |
| --- | --- | --- | --- | --- | --- | --- | --- | --- |
|  | Unrelated Sample | 3^rd^ degree Sample | 2^nd^ degree Sample | 1^st^ degree Sample | Unrelated Sample | 3^rd^ degree Sample | 2^nd^ degree Sample | 1^st^ degree Sample |
| Leg predicted mass (left) | 0.072 | 0.106 | 0.109 | 0.156 | 0.051 | 0.132 | 0.125 | 0.168 |
| Arm fat-free mass (right) | 0.072 | 0.106 | 0.109 | 0.156 | 0.019 | 0.113 | 0.109 | 0.151 |
| Arm fat-free mass (left) | 0.072 | 0.106 | 0.109 | 0.155 | 0.03 | 0.114 | 0.115 | 0.162 |
| Arm predicted mass (right) | 0.071 | 0.105 | 0.108 | 0.155 | 0.032 | 0.109 | 0.107 | 0.15 |
| Leg fat-free mass (right) | 0.071 | 0.105 | 0.108 | 0.155 | 0.028 | 0.133 | 0.132 | 0.172 |
| Arm predicted mass (left) | 0.071 | 0.105 | 0.108 | 0.154 | 0.032 | 0.113 | 0.115 | 0.163 |
| Leg predicted mass (right) | 0.071 | 0.105 | 0.108 | 0.155 | 0.026 | 0.131 | 0.129 | 0.169 |
| Impedance of whole body | 0.071 | 0.105 | 0.108 | 0.154 | 0.082 | 0.151 | 0.143 | 0.222 |
| Impedance of leg (left) | 0.067 | 0.099 | 0.102 | 0.145 | 0.116 | 0.158 | 0.187 | 0.26 |
| Impedance of leg (right) | 0.066 | 0.098 | 0.101 | 0.144 | 0.075 | 0.151 | 0.181 | 0.269 |
| Impedance of arm (right) | 0.066 | 0.097 | 0.1 | 0.142 | 0.059 | 0.118 | 0.115 | 0.179 |
| Whole body water mass | 0.077 | 0.114 | 0.117 | 0.167 | 0.01 | 0.125 | 0.123 | 0.174 |
| Whole body fat-free mass | 0.077 | 0.114 | 0.117 | 0.17 | 0.023 | 0.125 | 0.123 | 0.175 |
| Basal metabolic rate | 0.076 | 0.113 | 0.116 | 0.165 | 0.026 | 0.136 | 0.136 | 0.195 |
| Trunk fat-free mass | 0.076 | 0.112 | 0.116 | 0.165 | 0.017 | 0.121 | 0.118 | 0.175 |
| Trunk predicted mass | 0.076 | 0.111 | 0.115 | 0.164 | 0.031 | 0.119 | 0.115 | 0.17 |
| Leg fat-free mass (left) | 0.072 | 0.106 | 0.109 | 0.156 | 0.017 | 0.134 | 0.129 | 0.172 |
| Qualifications: None of the above | 0.028 | 0.043 | 0.044 | 0.063 | 0.025 | 0.154 | 0.108 | 0.114 |
| Blood clot, DVT, bronchitis, emphysema,  asthma, rhinitis, eczema, allergy diagnosed by  doctor: Hayfever, allergic rhinitis or eczema | 0.02 | 0.03 | 0.031 | 0.045 | 0.028 | 0.043 | 0.064 | 0.123 |
| Sensitivity / hurt feelings | 0.018 | 0.028 | 0.028 | 0.041 | 0.029 | 0.053 | 0.06 | 0.114 |
| Vascular/heart problems diagnosed by doctor:  High blood pressure | 0.035 | 0.051 | 0.053 | 0.077 | 0.081 | 0.104 | 0.072 | 0.089 |
| Arm fat percentage (left) | 0.061 | 0.09 | 0.092 | 0.136 | 0.039 | 0.104 | 0.101 | 0.21 |
| Leg fat mass (right) | 0.064 | 0.094 | 0.096 | 0.141 | 0.028 | 0.118 | 0.097 | 0.257 |
| Whole body fat mass | 0.065 | 0.095 | 0.097 | 0.143 | 0.057 | 0.17 | 0.154 | 0.304 |
| Leg fat mass (left) | 0.064 | 0.095 | 0.097 | 0.143 | 0.027 | 0.115 | 0.094 | 0.255 |
| Arm fat mass (right) | 0.064 | 0.095 | 0.097 | 0.142 | 0.067 | 0.133 | 0.053 | 0.297 |
| Body fat percentage | 0.06 | 0.088 | 0.092 | 0.135 | 0.025 | 0.113 | 0.108 | 0.199 |
| Arm fat percentage (right) | 0.06 | 0.089 | 0.091 | 0.134 | 0.044 | 0.103 | 0.1 | 0.207 |
| Pulse rate, automated reading | 0.043 | 0.064 | 0.066 | 0.096 | 0.046 | 0.052 | 0.104 | 0.149 |
| Past tobacco smoking | 0.027 | 0.04 | 0.04 | 0.059 | 0.022 | 0.106 | 0.093 | 0.166 |
| Alcohol intake frequency. | 0.024 | 0.036 | 0.038 | 0.055 | 0.036 | 0.102 | 0.128 | 0.226 |
| Comparative body size at age 10 | 0.04 | 0.06 | 0.061 | 0.09 | 0.037 | 0.094 | 0.074 | 0.112 |
| Mood swings | 0.021 | 0.031 | 0.032 | 0.047 | 0.019 | 0.083 | 0.048 | 0.135 |
| Miserableness | 0.019 | 0.028 | 0.029 | 0.043 | 0.004 | 0.065 | 0.061 | 0.116 |
| Irritability | 0.02 | 0.03 | 0.03 | 0.045 | 0.044 | 0.054 | 0.022 | 0.076 |
| Fed-up feelings | 0.021 | 0.032 | 0.033 | 0.047 | 0.044 | 0.08 | 0.041 | 0.098 |
| Nervous feelings | 0.019 | 0.029 | 0.03 | 0.043 | 0.033 | 0.061 | 0.062 | 0.096 |
| Worry too long after embarrassment | 0.019 | 0.028 | 0.029 | 0.043 | 0.01 | 0.023 | 0.048 | 0.098 |
| Neuroticism score | 0.034 | 0.049 | 0.048 | 0.071 | 0.009 | 0.097 | 0.082 | 0.132 |
| Sitting height | 0.083 | 0.122 | 0.127 | 0.181 | 0.065 | 0.16 | 0.171 | 0.245 |
| Ever smoked | 0.022 | 0.033 | 0.033 | 0.048 | 0.031 | 0.077 | 0.059 | 0.149 |
| Body mass index (BMI) | 0.068 | 0.1 | 0.105 | 0.149 | 0.078 | 0.181 | 0.176 | 0.309 |
| Weight | 0.072 | 0.107 | 0.11 | 0.157 | 0.085 | 0.188 | 0.188 | 0.303 |
| Diastolic blood pressure, automated reading | 0.039 | 0.057 | 0.058 | 0.086 | 0.003 | 0.124 | 0.102 | 0.179 |
| Systolic blood pressure, automated reading | 0.037 | 0.054 | 0.055 | 0.081 | 0.026 | 0.133 | 0.069 | 0.103 |
| Waist circumference | 0.056 | 0.082 | 0.086 | 0.123 | 0.063 | 0.136 | 0.152 | 0.235 |
| Hip circumference | 0.06 | 0.089 | 0.092 | 0.132 | 0.05 | 0.164 | 0.155 | 0.291 |
| Standing height | 0.118 | 0.172 | 0.179 | 0.25 | 0.116 | 0.182 | 0.214 | 0.313 |
| Qualifications: College or University degree | 0.048 | 0.072 | 0.073 | 0.105 | 0.093 | 0.163 | 0.185 | 0.249 |
| Heel bone mineral density (BMD) T-score, automated | 0.08 | 0.115 | 0.11 | 0.16 | 0.105 | 0.168 | 0.147 | 0.271 |

**Supplementary Table 7. Detailed result for prevalence of 12 dichotomous traits according to the percentile value of PRS.**

| Design | Analysis | Trait name | 10 | 20 | 30 | 40 | 50 | 60 | 70 | 80 | 90 | 92 | 95 | 99 | 99.9 | 99.95 | 99.99 |
| --- | --- | --- | --- | --- | --- | --- | --- | --- | --- | --- | --- | --- | --- | --- | --- | --- | --- |
| Large-scale design | Unrelated sample | Qualifications: None of the above | 0.176 | 0.185 | 0.192 | 0.202 | 0.212 | 0.221 | 0.233 | 0.248 | 0.279 | 0.283 | 0.294 | 0.34 | 0.293 | 0.379 | 0.5 |
|  |  | Blood clot, DVT, bronchitis, emphysema, asthma, rhinitis, eczema,  allergy diagnosed by doctor: Hayfever, allergic rhinitis or eczema | 0.241 | 0.25 | 0.259 | 0.266 | 0.275 | 0.287 | 0.299 | 0.315 | 0.335 | 0.345 | 0.355 | 0.39 | 0.586 | 0.586 | 0.5 |
|  |  | Sensitivity / hurt feelings | 0.565 | 0.572 | 0.578 | 0.586 | 0.594 | 0.599 | 0.605 | 0.619 | 0.635 | 0.636 | 0.641 | 0.681 | 0.719 | 0.862 | 0.667 |
|  |  | Vascular/heart problems diagnosed by doctor: High blood pressure | 0.284 | 0.295 | 0.306 | 0.317 | 0.33 | 0.345 | 0.362 | 0.385 | 0.413 | 0.429 | 0.443 | 0.506 | 0.655 | 0.621 | 0.833 |
|  |  | Mood swings | 0.456 | 0.464 | 0.472 | 0.479 | 0.49 | 0.499 | 0.51 | 0.518 | 0.537 | 0.541 | 0.56 | 0.566 | 0.544 | 0.655 | 0.833 |
|  |  | Miserableness | 0.434 | 0.442 | 0.448 | 0.457 | 0.463 | 0.471 | 0.483 | 0.494 | 0.51 | 0.52 | 0.543 | 0.548 | 0.667 | 0.759 | 0.667 |
|  |  | Irritability | 0.288 | 0.293 | 0.301 | 0.308 | 0.317 | 0.324 | 0.334 | 0.348 | 0.365 | 0.376 | 0.387 | 0.432 | 0.518 | 0.536 | 0.833 |
|  |  | Fed-up feelings | 0.411 | 0.42 | 0.426 | 0.434 | 0.443 | 0.453 | 0.466 | 0.481 | 0.497 | 0.506 | 0.528 | 0.578 | 0.737 | 0.724 | 0.667 |
|  |  | Nervous feelings | 0.245 | 0.251 | 0.258 | 0.266 | 0.272 | 0.279 | 0.289 | 0.298 | 0.319 | 0.325 | 0.333 | 0.381 | 0.368 | 0.414 | 0.667 |
|  |  | Worry too long after embarrassment | 0.487 | 0.497 | 0.505 | 0.512 | 0.522 | 0.531 | 0.54 | 0.55 | 0.564 | 0.574 | 0.583 | 0.607 | 0.768 | 0.786 | 0.667 |
|  |  | Ever smoked | 0.613 | 0.62 | 0.629 | 0.636 | 0.643 | 0.652 | 0.664 | 0.679 | 0.688 | 0.697 | 0.704 | 0.737 | 0.792 | 0.778 | 0.5 |
|  |  | Qualifications: College or University degree | 0.35 | 0.367 | 0.383 | 0.399 | 0.419 | 0.437 | 0.463 | 0.495 | 0.536 | 0.555 | 0.579 | 0.656 | 0.776 | 0.828 | 0.833 |
| Small-scale design | Unrelated sample | Qualifications: None of the above | 0.158 | 0.158 | 0.157 | 0.157 | 0.164 | 0.158 | 0.16 | 0.145 | 0.13 | 0.125 | 0.14 | 0.1 |  |  |  |
|  |  | Blood clot, DVT, bronchitis, emphysema, asthma, rhinitis, eczema,  allergy diagnosed by doctor: Hayfever, allergic rhinitis or eczema | 0.232 | 0.236 | 0.229 | 0.233 | 0.236 | 0.235 | 0.237 | 0.225 | 0.26 | 0.25 | 0.2 | 0.3 |  |  |  |
|  |  | Sensitivity / hurt feelings | 0.582 | 0.581 | 0.596 | 0.585 | 0.586 | 0.588 | 0.613 | 0.64 | 0.65 | 0.662 | 0.68 | 0.6 |  |  |  |
|  |  | Vascular/heart problems diagnosed by doctor: High blood pressure | 0.277 | 0.285 | 0.293 | 0.302 | 0.286 | 0.302 | 0.287 | 0.295 | 0.32 | 0.3 | 0.36 | 0.4 |  |  |  |
|  |  | Mood swings | 0.449 | 0.441 | 0.433 | 0.443 | 0.446 | 0.432 | 0.443 | 0.43 | 0.41 | 0.438 | 0.42 | 0.4 |  |  |  |
|  |  | Miserableness | 0.427 | 0.43 | 0.423 | 0.435 | 0.426 | 0.415 | 0.407 | 0.42 | 0.44 | 0.462 | 0.48 | 0.6 |  |  |  |
|  |  | Irritability | 0.272 | 0.266 | 0.273 | 0.275 | 0.286 | 0.272 | 0.277 | 0.27 | 0.29 | 0.312 | 0.38 | 0.6 |  |  |  |
|  |  | Fed-up feelings | 0.443 | 0.439 | 0.446 | 0.462 | 0.468 | 0.485 | 0.507 | 0.515 | 0.49 | 0.475 | 0.42 | 0.4 |  |  |  |
|  |  | Nervous feelings | 0.264 | 0.252 | 0.25 | 0.26 | 0.256 | 0.235 | 0.257 | 0.28 | 0.34 | 0.338 | 0.34 | 0.4 |  |  |  |
|  |  | Worry too long after embarrassment | 0.469 | 0.471 | 0.476 | 0.485 | 0.478 | 0.5 | 0.497 | 0.51 | 0.62 | 0.6 | 0.48 | 0.7 |  |  |  |
|  |  | Ever smoked | 0.608 | 0.611 | 0.613 | 0.605 | 0.59 | 0.595 | 0.597 | 0.59 | 0.63 | 0.638 | 0.68 | 0.6 |  |  |  |
|  |  | Qualifications: College or University degree | 0.348 | 0.355 | 0.361 | 0.362 | 0.378 | 0.392 | 0.417 | 0.44 | 0.45 | 0.462 | 0.48 | 0.5 |  |  |  |
|  | First degree sample | Qualifications: None of the above | 0.309 | 0.322 | 0.34 | 0.355 | 0.378 | 0.395 | 0.4 | 0.39 | 0.38 | 0.388 | 0.38 | 0.6 |  |  |  |
|  |  | Blood clot, DVT, bronchitis, emphysema, asthma, rhinitis, eczema,  allergy diagnosed by doctor: Hayfever, allergic rhinitis or eczema | 0.233 | 0.238 | 0.244 | 0.255 | 0.264 | 0.285 | 0.307 | 0.345 | 0.38 | 0.338 | 0.42 | 0.4 |  |  |  |
|  |  | Sensitivity / hurt feelings | 0.576 | 0.589 | 0.603 | 0.61 | 0.614 | 0.61 | 0.603 | 0.61 | 0.65 | 0.65 | 0.64 | 0.6 |  |  |  |
|  |  | Vascular/heart problems diagnosed by doctor: High blood pressure | 0.284 | 0.294 | 0.309 | 0.327 | 0.338 | 0.345 | 0.343 | 0.345 | 0.37 | 0.375 | 0.42 | 0.5 |  |  |  |
|  |  | Mood swings | 0.488 | 0.495 | 0.507 | 0.53 | 0.542 | 0.56 | 0.597 | 0.62 | 0.64 | 0.662 | 0.72 | 0.8 |  |  |  |
|  |  | Miserableness | 0.434 | 0.449 | 0.463 | 0.48 | 0.488 | 0.502 | 0.497 | 0.475 | 0.51 | 0.5 | 0.5 | 0.4 |  |  |  |
|  |  | Irritability | 0.261 | 0.266 | 0.274 | 0.272 | 0.282 | 0.295 | 0.297 | 0.295 | 0.29 | 0.275 | 0.28 | 0.2 |  |  |  |
|  |  | Fed-up feelings | 0.387 | 0.395 | 0.409 | 0.428 | 0.434 | 0.438 | 0.46 | 0.455 | 0.53 | 0.538 | 0.54 | 0.5 |  |  |  |
|  |  | Nervous feelings | 0.237 | 0.248 | 0.257 | 0.265 | 0.276 | 0.288 | 0.287 | 0.295 | 0.34 | 0.338 | 0.28 | 0.6 |  |  |  |
|  |  | Worry too long after embarrassment | 0.471 | 0.482 | 0.493 | 0.495 | 0.5 | 0.498 | 0.497 | 0.525 | 0.55 | 0.562 | 0.62 | 0.7 |  |  |  |
|  |  | Ever smoked | 0.587 | 0.591 | 0.61 | 0.628 | 0.642 | 0.642 | 0.653 | 0.69 | 0.69 | 0.65 | 0.62 | 0.6 |  |  |  |
|  |  | Qualifications: College or University degree | 0.232 | 0.236 | 0.253 | 0.257 | 0.276 | 0.312 | 0.34 | 0.42 | 0.52 | 0.538 | 0.6 | 0.8 |  |  |  |

**Supplementary Table 8. Detailed result for odds ratio of 12 dichotomous traits according to the percentile value of PRS.**

| Design | Analysis | Trait name | 10 | 20 | 30 | 40 | 50 | 60 | 70 | 80 | 90 | 92 | 95 | 99 | 99.9 | 99.95 | 99.99 |
| --- | --- | --- | --- | --- | --- | --- | --- | --- | --- | --- | --- | --- | --- | --- | --- | --- | --- |
| Large-scale design | Unrelated sample | Qualifications: None of the above | 1.082 | 1.151 | 1.208 | 1.286 | 1.363 | 1.443 | 1.544 | 1.678 | 1.961 | 2.004 | 2.116 | 2.62 | 2.106 | 3.104 | 5.079 |
|  |  | Blood clot, DVT, bronchitis, emphysema, asthma, rhinitis, eczema,  allergy diagnosed by doctor: Hayfever, allergic rhinitis or eczema | 1.048 | 1.102 | 1.152 | 1.199 | 1.254 | 1.326 | 1.405 | 1.52 | 1.661 | 1.737 | 1.816 | 2.109 | 4.675 | 4.675 | 3.3 |
|  |  | Sensitivity / hurt feelings | 1.04 | 1.068 | 1.096 | 1.132 | 1.17 | 1.196 | 1.223 | 1.299 | 1.392 | 1.4 | 1.429 | 1.711 | 2.049 | 4.998 | 1.599 |
|  |  | Vascular/heart problems diagnosed by doctor: High blood pressure | 1.072 | 1.132 | 1.192 | 1.254 | 1.328 | 1.423 | 1.533 | 1.69 | 1.898 | 2.029 | 2.151 | 2.766 | 5.129 | 4.418 | 13.498 |
|  |  | Mood swings | 1.041 | 1.075 | 1.107 | 1.143 | 1.19 | 1.238 | 1.291 | 1.334 | 1.437 | 1.464 | 1.58 | 1.621 | 1.48 | 2.358 | 6.205 |
|  |  | Miserableness | 1.042 | 1.076 | 1.104 | 1.143 | 1.174 | 1.213 | 1.27 | 1.325 | 1.418 | 1.471 | 1.614 | 1.651 | 2.72 | 4.274 | 2.72 |
|  |  | Irritability | 1.039 | 1.066 | 1.109 | 1.144 | 1.191 | 1.23 | 1.288 | 1.375 | 1.475 | 1.551 | 1.622 | 1.958 | 2.763 | 2.968 | 12.86 |
|  |  | Fed-up feelings | 1.042 | 1.08 | 1.109 | 1.145 | 1.188 | 1.236 | 1.304 | 1.383 | 1.477 | 1.53 | 1.673 | 2.05 | 4.182 | 3.921 | 2.987 |
|  |  | Nervous feelings | 1.039 | 1.076 | 1.115 | 1.162 | 1.196 | 1.243 | 1.303 | 1.36 | 1.5 | 1.546 | 1.601 | 1.976 | 1.871 | 2.264 | 6.415 |
|  |  | Worry too long after embarrassment | 1.038 | 1.08 | 1.115 | 1.146 | 1.194 | 1.234 | 1.282 | 1.337 | 1.414 | 1.47 | 1.524 | 1.689 | 3.613 | 4.005 | 2.185 |
|  |  | Ever smoked | 1.051 | 1.082 | 1.123 | 1.157 | 1.194 | 1.239 | 1.307 | 1.4 | 1.462 | 1.525 | 1.576 | 1.859 | 2.531 | 2.32 | 0.663 |
|  |  | Qualifications: College or University degree | 1.081 | 1.163 | 1.247 | 1.33 | 1.445 | 1.56 | 1.729 | 1.968 | 2.321 | 2.502 | 2.762 | 3.831 | 6.949 | 9.635 | 10.037 |
| Small-scale design | Unrelated sample | Qualifications: None of the above | 0.976 | 0.974 | 0.972 | 0.968 | 1.022 | 0.974 | 0.993 | 0.884 | 0.779 | 0.744 | 0.848 | 0.579 |  |  |  |
|  |  | Blood clot, DVT, bronchitis, emphysema, asthma, rhinitis, eczema,  allergy diagnosed by doctor: Hayfever, allergic rhinitis or eczema | 1.036 | 1.059 | 1.015 | 1.042 | 1.058 | 1.052 | 1.062 | 0.994 | 1.203 | 1.142 | 0.856 | 1.468 |  |  |  |
|  |  | Sensitivity / hurt feelings | 1.026 | 1.022 | 1.085 | 1.038 | 1.042 | 1.048 | 1.168 | 1.309 | 1.367 | 1.445 | 1.564 | 1.104 |  |  |  |
|  |  | Vascular/heart problems diagnosed by doctor: High blood pressure | 1.013 | 1.056 | 1.097 | 1.145 | 1.061 | 1.149 | 1.065 | 1.109 | 1.247 | 1.136 | 1.49 | 1.766 |  |  |  |
|  |  | Mood swings | 1.004 | 0.973 | 0.94 | 0.981 | 0.992 | 0.939 | 0.981 | 0.93 | 0.856 | 0.958 | 0.892 | 0.821 |  |  |  |
|  |  | Miserableness | 0.971 | 0.984 | 0.956 | 1.004 | 0.968 | 0.925 | 0.894 | 0.944 | 1.025 | 1.122 | 1.204 | 1.956 |  |  |  |
|  |  | Irritability | 1.043 | 1.012 | 1.046 | 1.057 | 1.117 | 1.044 | 1.066 | 1.031 | 1.139 | 1.267 | 1.709 | 4.182 |  |  |  |
|  |  | Fed-up feelings | 1.01 | 0.991 | 1.019 | 1.087 | 1.115 | 1.194 | 1.302 | 1.346 | 1.218 | 1.147 | 0.918 | 0.845 |  |  |  |
|  |  | Nervous feelings | 1.073 | 1.008 | 0.995 | 1.048 | 1.027 | 0.917 | 1.03 | 1.16 | 1.537 | 1.52 | 1.537 | 1.989 |  |  |  |
|  |  | Worry too long after embarrassment | 0.988 | 0.997 | 1.015 | 1.053 | 1.024 | 1.119 | 1.104 | 1.164 | 1.825 | 1.678 | 1.033 | 2.61 |  |  |  |
|  |  | Ever smoked | 0.995 | 1.01 | 1.016 | 0.983 | 0.924 | 0.943 | 0.95 | 0.924 | 1.093 | 1.129 | 1.364 | 0.963 |  |  |  |
|  |  | Qualifications: College or University degree | 1.017 | 1.05 | 1.079 | 1.08 | 1.159 | 1.232 | 1.362 | 1.498 | 1.56 | 1.641 | 1.76 | 1.907 |  |  |  |
|  | First degree sample | Qualifications: None of the above | 1.121 | 1.194 | 1.292 | 1.381 | 1.525 | 1.638 | 1.673 | 1.604 | 1.538 | 1.587 | 1.538 | 3.763 |  |  |  |
|  |  | Blood clot, DVT, bronchitis, emphysema, asthma, rhinitis, eczema,  allergy diagnosed by doctor: Hayfever, allergic rhinitis or eczema | 1.067 | 1.092 | 1.133 | 1.2 | 1.257 | 1.397 | 1.55 | 1.846 | 2.148 | 1.785 | 2.538 | 2.336 |  |  |  |
|  |  | Sensitivity / hurt feelings | 1.057 | 1.116 | 1.183 | 1.219 | 1.24 | 1.219 | 1.185 | 1.219 | 1.447 | 1.447 | 1.386 | 1.169 |  |  |  |
|  |  | Vascular/heart problems diagnosed by doctor: High blood pressure | 1.069 | 1.119 | 1.201 | 1.305 | 1.373 | 1.417 | 1.406 | 1.417 | 1.58 | 1.614 | 1.948 | 2.69 |  |  |  |
|  |  | Mood swings | 1.083 | 1.114 | 1.17 | 1.282 | 1.345 | 1.447 | 1.682 | 1.855 | 2.021 | 2.231 | 2.923 | 4.547 |  |  |  |
|  |  | Miserableness | 1.014 | 1.075 | 1.138 | 1.219 | 1.258 | 1.333 | 1.303 | 1.194 | 1.374 | 1.32 | 1.32 | 0.88 |  |  |  |
|  |  | Irritability | 0.995 | 1.022 | 1.065 | 1.051 | 1.106 | 1.179 | 1.188 | 1.179 | 1.151 | 1.068 | 1.095 | 0.704 |  |  |  |
|  |  | Fed-up feelings | 1.003 | 1.039 | 1.099 | 1.192 | 1.22 | 1.237 | 1.355 | 1.328 | 1.794 | 1.849 | 1.867 | 1.591 |  |  |  |
|  |  | Nervous feelings | 1.009 | 1.071 | 1.127 | 1.174 | 1.241 | 1.314 | 1.308 | 1.362 | 1.677 | 1.658 | 1.266 | 4.883 |  |  |  |
|  |  | Worry too long after embarrassment | 1.033 | 1.081 | 1.127 | 1.137 | 1.16 | 1.148 | 1.144 | 1.282 | 1.418 | 1.491 | 1.892 | 2.706 |  |  |  |
|  |  | Ever smoked | 1.084 | 1.105 | 1.194 | 1.291 | 1.369 | 1.372 | 1.439 | 1.7 | 1.7 | 1.418 | 1.246 | 1.146 |  |  |  |
|  |  | Qualifications: College or University degree | 1.072 | 1.097 | 1.2 | 1.224 | 1.352 | 1.612 | 1.826 | 2.567 | 3.841 | 4.12 | 5.318 | 14.182 |  |  |  |

**Supplementary Table 9. Fifty traits according to decreasing order of narrow-sense heritability for sensitivity analysis.**

| Trait order (t) | Trait name | Narrow-sense heritability |
| --- | --- | --- |
| 1 | Standing height | 0.4 |
| 2 | Sitting height | 0.28 |
| 3 | Heel bone mineral density (BMD) T-score, automated | 0.27 |
| 4 | Whole body water mass | 0.26 |
| 5 | Whole body fat-free mass | 0.26 |
| 6 | Basal metabolic rate | 0.26 |
| 7 | Trunk fat-free mass | 0.26 |
| 8 | Trunk predicted mass | 0.26 |
| 9 | Leg predicted mass (left) | 0.24 |
| 10 | Arm fat-free mass (right) | 0.24 |
| 11 | Arm fat-free mass (left) | 0.24 |
| 12 | Arm predicted mass (right) | 0.24 |
| 13 | Leg fat-free mass (right) | 0.24 |
| 14 | Arm predicted mass (left) | 0.24 |
| 15 | Leg predicted mass (right) | 0.24 |
| 16 | Impedance of whole body | 0.24 |
| 17 | Leg fat-free mass (left) | 0.24 |
| 18 | Weight | 0.24 |
| 19 | Body mass index (BMI) | 0.23 |
| 20 | Impedance of leg (left) | 0.22 |
| 21 | Impedance of leg (right) | 0.22 |
| 22 | Impedance of arm (right) | 0.22 |
| 23 | Leg fat mass (right) | 0.22 |
| 24 | Whole body fat mass | 0.22 |
| 25 | Leg fat mass (left) | 0.22 |
| 26 | Arm fat mass (right) | 0.22 |
| 27 | Arm fat percentage (left) | 0.21 |
| 28 | Body fat percentage | 0.2 |
| 29 | Arm fat percentage (right) | 0.2 |
| 30 | Hip circumference | 0.2 |
| 31 | Waist circumference | 0.19 |
| 32 | Qualifications: College or University degree | 0.16 |
| 33 | Pulse rate, automated reading | 0.15 |
| 34 | Comparative body size at age 10 | 0.13 |
| 35 | Diastolic blood pressure, automated reading | 0.13 |
| 36 | "Vascular/heart problems diagnosed by doctor:  High blood pressure" | 0.12 |
| 37 | Systolic blood pressure, automated reading | 0.12 |
| 38 | Neuroticism score | 0.11 |
| 39 | Qualifications: None of the above | 0.1 |
| 40 | Past tobacco smoking | 0.09 |
| 41 | Alcohol intake frequency. | 0.08 |
| 42 | "Blood clot, DVT, bronchitis, emphysema,  asthma, rhinitis, eczema, allergy diagnosed by  doctor: Hayfever, allergic rhinitis or eczema" | 0.07 |
| 43 | Mood swings | 0.07 |
| 44 | Irritability | 0.07 |
| 45 | Fed-up feelings | 0.07 |
| 46 | Ever smoked | 0.07 |
| 47 | Sensitivity / hurt feelings | 0.06 |
| 48 | Miserableness | 0.06 |
| 49 | Nervous feelings | 0.06 |
| 50 | Worry too long after embarrassment | 0.06 |

Estimated narrow-sense heritability for each trait is adopted from Neale et al.

**Supplementary Table 10. Prediction accuracies of various methods with and without phenotypic information of ungenotyped relatives of the target sample.**

| Trait name | PRS_5000_ | PRS_2000_ | GBLUP_2000_ | GBLUP_5000_ | HBLUP_5000_ | ABLUP_5000_ |
| --- | --- | --- | --- | --- | --- | --- |
| Leg predicted mass (left) | 0.356 | 0.236 | 0.240 | 0.366 | 0.310 | 0.221 |
| Arm fat-free mass (right) | 0.331 | 0.204 | 0.207 | 0.339 | 0.268 | 0.188 |
| Arm fat-free mass (left) | 0.328 | 0.185 | 0.185 | 0.339 | 0.253 | 0.205 |
| Arm predicted mass (right) | 0.335 | 0.204 | 0.206 | 0.340 | 0.266 | 0.186 |
| Leg fat-free mass (right) | 0.332 | 0.236 | 0.247 | 0.336 | 0.302 | 0.224 |
| Arm predicted mass (left) | 0.333 | 0.191 | 0.191 | 0.343 | 0.256 | 0.207 |
| Leg predicted mass (right) | 0.331 | 0.236 | 0.247 | 0.334 | 0.300 | 0.222 |
| Impedance of whole body | 0.313 | 0.233 | 0.239 | 0.309 | 0.271 | 0.185 |
| Impedance of leg (left) | 0.270 | 0.170 | 0.177 | 0.268 | 0.218 | 0.166 |
| Impedance of leg (right) | 0.275 | 0.175 | 0.184 | 0.278 | 0.224 | 0.174 |
| Impedance of arm (right) | 0.325 | 0.223 | 0.218 | 0.321 | 0.257 | 0.200 |
| Whole body water mass | 0.337 | 0.247 | 0.257 | 0.334 | 0.302 | 0.209 |
| Whole body fat-free mass | 0.381 | 0.271 | 0.269 | 0.390 | 0.345 | 0.244 |
| Basal metabolic rate | 0.339 | 0.249 | 0.260 | 0.337 | 0.308 | 0.221 |
| Trunk fat-free mass | 0.334 | 0.186 | 0.189 | 0.339 | 0.251 | 0.187 |
| Trunk predicted mass | 0.332 | 0.184 | 0.187 | 0.337 | 0.250 | 0.186 |
| Leg fat-free mass (left) | 0.357 | 0.234 | 0.238 | 0.366 | 0.310 | 0.222 |
| Qualifications: None of the above | 0.209 | 0.141 | 0.138 | 0.200 | 0.114 | 0.025 |
| "Blood clot, DVT, bronchitis, emphysema,  asthma, rhinitis, eczema, allergy diagnosed by  doctor: Hayfever, allergic rhinitis or eczema" | 0.155 | 0.089 | 0.090 | 0.154 | 0.107 | 0.066 |
| Sensitivity / hurt feelings | 0.113 | 0.050 | 0.051 | 0.114 | 0.059 | 0.039 |
| "Vascular/heart problems diagnosed by doctor:  High blood pressure" | 0.142 | 0.092 | 0.091 | 0.135 | 0.092 | 0.029 |
| Arm fat percentage (left) | 0.275 | 0.167 | 0.167 | 0.288 | 0.231 | 0.211 |
| Leg fat mass (right) | 0.244 | 0.199 | 0.194 | 0.244 | 0.210 | 0.167 |
| Whole body fat mass | 0.279 | 0.157 | 0.157 | 0.287 | 0.214 | 0.214 |
| Leg fat mass (left) | 0.274 | 0.172 | 0.171 | 0.282 | 0.236 | 0.212 |
| Arm fat mass (right) | 0.266 | 0.155 | 0.159 | 0.280 | 0.231 | 0.224 |
| Body fat percentage | 0.276 | 0.203 | 0.209 | 0.284 | 0.223 | 0.185 |
| Arm fat percentage (right) | 0.286 | 0.177 | 0.176 | 0.298 | 0.242 | 0.211 |
| Pulse rate, automated reading | 0.104 | 0.099 | 0.099 | 0.109 | 0.137 | 0.091 |
| Past tobacco smoking | 0.211 | 0.169 | 0.168 | 0.214 | 0.173 | 0.090 |
| Alcohol intake frequency. | 0.269 | 0.169 | 0.162 | 0.240 | 0.190 | 0.126 |
| Comparative body size at age 10 | 0.101 | 0.040 | 0.044 | 0.102 | 0.079 | 0.099 |
| Mood swings | 0.184 | 0.128 | 0.124 | 0.182 | 0.142 | 0.102 |
| Miserableness | 0.083 | 0.072 | 0.070 | 0.084 | 0.080 | 0.065 |
| Irritability | 0.042 | 0.027 | 0.021 | 0.042 | 0.055 | 0.013 |
| Fed-up feelings | 0.088 | 0.046 | 0.043 | 0.088 | 0.031 | 0.026 |
| Nervous feelings | 0.100 | 0.035 | 0.037 | 0.102 | 0.080 | 0.086 |
| Worry too long after embarrassment | 0.091 | 0.038 | 0.041 | 0.091 | 0.057 | 0.033 |
| Neuroticism score | 0.123 | 0.059 | 0.060 | 0.131 | 0.098 | 0.062 |
| Sitting height | 0.315 | 0.191 | 0.201 | 0.320 | 0.252 | 0.200 |
| Ever smoked | 0.163 | 0.079 | 0.080 | 0.162 | 0.118 | 0.097 |
| Body mass index (BMI) | 0.305 | 0.234 | 0.236 | 0.313 | 0.276 | 0.180 |
| Weight | 0.325 | 0.153 | 0.169 | 0.341 | 0.252 | 0.236 |
| Diastolic blood pressure, automated reading | 0.224 | 0.116 | 0.115 | 0.221 | 0.125 | 0.041 |
| Systolic blood pressure, automated reading | 0.188 | 0.125 | 0.121 | 0.181 | 0.135 | 0.056 |
| Waist circumference | 0.325 | 0.200 | 0.206 | 0.325 | 0.232 | 0.182 |
| Hip circumference | 0.293 | 0.176 | 0.172 | 0.302 | 0.219 | 0.181 |
| Standing height | 0.506 | 0.356 | 0.371 | 0.535 | 0.461 | 0.387 |
| Qualifications: College or University degree | 0.245 | 0.160 | 0.154 | 0.234 | 0.176 | 0.093 |
| Heel bone mineral density (BMD) T-score, automated | 0.261 | 0.188 | 0.194 | 0.272 | 0.225 | 0.165 |

The subscript in the name of each method represents the number of individuals in the discovery sample. PRS_5000_: Polygenic Risk Score with 5000 genotyped individuals; GBLUP_5000_: Best Linear Unbiased Prediction integrated with Genomic Relationship Matrix with genotyped individuals. PRS_2000_: Polygenic Risk Score with 2000 genotyped individuals only. GBLUP_2000_: Best Linear Unbiased Prediction integrated with Genomic Relationship Matrix with 2000 genotyped individuals only. HBLUP_5000_ and ABLUP_5000_: Best Linear Unbiased Prediction with genomic-pedigree relationship **H**-matrix and pedigree-relationship matrix including 2000 genotyped and 3000 ungenotyped individuals in the discovery sample.

**Supplementary Table 11. The average number of individuals per family.**

| Trait name | 3rd degree | 2nd degree | 1st degree |
| --- | --- | --- | --- |
| Qualifications: None of the above | 5.889 | 2.321 | 2.742 |
| Past tobacco smoking | 5.889 | 2.321 | 2.742 |
| Alcohol intake frequency. | 5.889 | 2.321 | 2.742 |
| Ever smoked | 5.889 | 2.321 | 2.742 |
| Qualifications: College or University degree | 5.889 | 2.322 | 2.743 |
| Sensitivity / hurt feelings | 5.889 | 2.321 | 2.742 |
| Mood swings | 5.889 | 2.322 | 2.743 |
| Miserableness | 5.889 | 2.322 | 2.743 |
| Irritability | 5.889 | 2.322 | 2.743 |
| Fed-up feelings | 5.889 | 2.322 | 2.743 |
| Nervous feelings | 5.889 | 2.322 | 2.742 |
| Worry too long after embarrassment | 5.889 | 2.322 | 2.743 |
| Neuroticism score | 5.889 | 2.322 | 2.742 |
| Leg predicted mass (left) | 5.889 | 2.322 | 2.743 |
| Arm fat-free mass (right) | 5.888 | 2.321 | 2.741 |
| Arm fat-free mass (left) | 5.887 | 2.321 | 2.741 |
| Arm predicted mass (right) | 5.889 | 2.322 | 2.742 |
| Leg fat-free mass (right) | 5.944 | 2.328 | 2.783 |
| Arm predicted mass (left) | 6.059 | 2.339 | 2.805 |
| Leg predicted mass (right) | 5.822 | 2.312 | 2.714 |
| Impedance of whole body | 6.055 | 2.337 | 2.808 |
| Impedance of leg (left) | 5.889 | 2.321 | 2.741 |
| Impedance of leg (right) | 5.889 | 2.321 | 2.741 |
| Impedance of arm (right) | 5.888 | 2.320 | 2.737 |
| Whole body water mass | 5.889 | 2.321 | 2.741 |
| Whole body fat-free mass | 5.891 | 2.321 | 2.741 |
| Basal metabolic rate | 5.888 | 2.321 | 2.741 |
| Trunk fat-free mass | 5.889 | 2.321 | 2.741 |
| Trunk predicted mass | 5.574 | 2.285 | 2.645 |
| Leg fat-free mass (left) | 5.322 | 2.240 | 2.585 |
| Blood clot, DVT, bronchitis, emphysema,  asthma, rhinitis, eczema, allergy diagnosed by  doctor: Hayfever, allergic rhinitis or eczema | 6.077 | 2.341 | 2.810 |
| Vascular/heart problems diagnosed by doctor: High blood pressure | 5.942 | 2.323 | 2.749 |
| Arm fat percentage (left) | 5.832 | 2.315 | 2.713 |
| Leg fat mass (right) | 5.874 | 2.322 | 2.744 |
| Whole body fat mass | 5.642 | 2.293 | 2.655 |
| Leg fat mass (left) | 5.869 | 2.318 | 2.740 |
| Arm fat mass (right) | 5.835 | 2.310 | 2.718 |
| Body fat percentage | 5.689 | 2.299 | 2.668 |
| Arm fat percentage (right) | 4.727 | 2.175 | 2.350 |
| Pulse rate, automated reading | 6.051 | 2.339 | 2.801 |
| Comparative body size at age 10 | 5.250 | 2.236 | 2.518 |
| Sitting height | 6.036 | 2.338 | 2.800 |
| Body mass index (BMI) | 6.042 | 2.339 | 2.800 |
| Weight | 5.415 | 2.261 | 2.581 |
| Diastolic blood pressure, automated reading | 5.415 | 2.261 | 2.581 |
| Systolic blood pressure, automated reading | 6.055 | 2.340 | 2.807 |
| Waist circumference | 6.059 | 2.339 | 2.804 |
| Hip circumference | 6.057 | 2.339 | 2.804 |
| Standing height | 5.944 | 2.328 | 2.783 |
| Heel bone mineral density (BMD) T-score, automated | 4.190 | 2.168 | 2.280 |
