# Supplementary material for: Efficient polygenic risk scores for biobank scale data by exploiting phenotypes from inferred relatives
Source: Nat Commun. 2020 Jun 17;11:3074. doi: 10.1038/s41467-020-16829-x (PMC7299943; doi:10.1038/s41467-020-16829-x)
Supplement: Supplementary file 3 — Reporting Summary [file 41467_2020_16829_MOESM3_ESM.pdf]

nature research

Corresponding author(s): S. Hong Lee

Last updated by author(s): May 3, 2020

Reporting Summary

Nature Research wishes to improve the reproducibility of the work that we publish. This form provides structure for consistency and transparency in reporting. For further information on Nature Research policies, see Authors & Referees and the Editorial Policy Checklist .

Statistics

For all statistical analyses, confirm that the following items are present in the figure legend, table legend, main text, or Methods section.

n/a	Confirmed	
☐	☒	The exact sample size (*n*) for each experimental group/condition, given as a discrete number and unit of measurement
☐	☒	A statement on whether measurements were taken from distinct samples or whether the same sample was measured repeatedly
☐	☒	The statistical test(s) used AND whether they are one- or two-sided<br>*Only common tests should be described solely by name; describe more complex techniques in the Methods section.*
☐	☒	A description of all covariates tested
☐	☒	A description of any assumptions or corrections, such as tests of normality and adjustment for multiple comparisons
☐	☒	A full description of the statistical parameters including central tendency (e.g. means) or other basic estimates (e.g. regression coefficient) AND variation (e.g. standard deviation) or associated estimates of uncertainty (e.g. confidence intervals)
☐	☒	For null hypothesis testing, the test statistic (e.g. *F*, *t*, *r*) with confidence intervals, effect sizes, degrees of freedom and *P* value noted<br>*Give P values as exact values whenever suitable.*
☒	☐	For Bayesian analysis, information on the choice of priors and Markov chain Monte Carlo settings
☒	☐	For hierarchical and complex designs, identification of the appropriate level for tests and full reporting of outcomes
☐	☒	Estimates of effect sizes (e.g. Cohen's *d*, Pearson's *r*), indicating how they were calculated

Our web collection on statistics for biologists contains articles on many of the points above.

Software and code

Policy information about availability of computer code

Data collection	The data used in this study were provided by the UK Biobank. No additional software was used to collect data.
Data analysis	We used MTG2 v2.15, R v3.5.2, PLINK v1.9, PRSice v2.1.11, KING v2.1, PRIMUS v1.9.0 and igraph v1.2.5 to analyze the data in this study.

For manuscripts utilizing custom algorithms or software that are central to the research but not yet described in published literature, software must be made available to editors/reviewers. We strongly encourage code deposition in a community repository (e.g. GitHub). See the Nature Research guidelines for submitting code & software for further information.

Data

Policy information about availability of data

All manuscripts must include a data availability statement. This statement should provide the following information, where applicable:

- Accession codes, unique identifiers, or web links for publicly available datasets
- A list of figures that have associated raw data
- A description of any restrictions on data availability

The raw genetic and phenotypic data that support the findings of this study are available from UK Biobank. UK Biobank data are publicly accessible through the procedure described in the webpage, http://www.ukbiobank.ac.uk/using-the-resource/. The source code for MTG2 version 2.15 is publicly available in https://sites.google.com/site/honglee0707/mtg2. The source data underlying Figs. 1–7 and Supplementary Figs. 1–8 are provided as a Source Data file. All other intermediate data generated in the downstream analyses in this study are available upon request.

Field-specific reporting

Please select the one below that is the best fit for your research. If you are not sure, read the appropriate sections before making your selection.

Life sciences study design

All studies must disclose on these points even when the disclosure is negative.

Sample size	We analyzed 50 traits from the UK biobank and sample size varies across traits, ranging from 237,191 (heel mineral density) to 407,938 (Alcohol intake frequency). No sample size calculation was needed as we used all available individuals. For PRS analyses, a sample size of 1000 already can have a sufficient power (PMID: 23555274) and the sample sizes used in this study are much higher than 1000.
Data exclusions	We performed analyses on a number of individuals limited to white British. The stringent quality control protocol was applied. SNPs were excluded following these criteria: INFO score< 0.6, MAF< 0.01, Hardy-Weinberg Equilibrium P-value< IE-7 and missingness > 5%, one SNP was randomly chosen to keep if there is duplicated SNPs. In terms of individuals filtering, calling rate of< 0.95 was applied. This filter retained 1,133,273 SNPs and 408,218 individuals remained. We removed ambiguous or duplicated SNPs. We only used SNPs from HapMap 3 due to its reliability and robust to bias to estimate narrow-sense heritability. Moreover, we excluded individual population whose first or second PC exceeded 6 standard deviations from the population mean. We calculated the discordance rate between imputed genotype of the first and the second release for each individual and for each SNP, individuals and SNPs with discordance rate larger than 0.05 were removed. We added one of any pairs with genomic relationship larger than 0.05 into our set of unrelated individuals. After these QC steps, 288,837 unrelated individuals and 1,130,918 SNPs remained. This quality control is well established and widely used in genetic studies e.g. GWAS and PRS. This quality control and flitering make sure that the results of the study are not biased due to low quality data.
Replication	We conducted 2 designs (large-scale and small-scale designs) with 50 traits using 4 levels of relatedness (unrelated, 3rd, 2nd and 1st degree relatives). The results of analyses were highly consistent and our finding is replicated across 50 traits.
Randomization	We used UK Biobank data, community samples. In the PRS analyses, we randomly selected individuals and divided into discovery and target dataset.
Blinding	Blinding was not relevant to our study, because the data were collected by UK Biobank and the ID of participants has been encrypted.

Reporting for specific materials, systems and methods

We require information from authors about some types of materials, experimental systems and methods used in many studies. Here, indicate whether each material, system or method listed is relevant to your study. If you are not sure if a list item applies to your research, read the appropriate section before selecting a response.

Materials & experimental systems

n/a	Involved in the study	
☒	☐	Antibodies
☒	☐	Eukaryotic cell lines
☒	☐	Palaeontology
☒	☐	Animals and other organisms
☐	☒	Human research participants
☒	☐	Clinical data

Methods

n/a	Involved in the study	
☒	☐	ChIP-seq
☒	☐	Flow cytometry
☒	☐	MRI-based neuroimaging

Human research participants

Policy information about studies involving human research participants

Population characteristics	UK Biobank data consist of 500,000 participants aged between 40-69 years in 2006-2010, with ~54% females and 46% males. Approximately 50,000 samples were genotyped on the UK BiLEVE Axiom array and the other 450,000 samples were genotyped on the UK Biobank Axiom array. Genotype were imputed to the whole genome level with IMPUTE3 using UK10K and 1000 Genome Phase 3 as the reference.
Recruitment	The recruitment includes a half million people from all around the UK who are currently aged 40-69 because the age group involves people at risk over the next few decades of developing a wide range of important diseases, conditions and covariates. The phenotype and genotype information is based on extensive baseline questionnaire and physical measures, as well as stored blood and urine samples that allow many different types of assay, incorporated with information from the UK National Health Service. The UK Biobank may not be fully representative of the general population, e.g. a low response rate with "healthy volunteer" selection bias (PMID 28641372). However, the effects of bias appear to be subtle, as reported in a recent study (PMID 26849114). In fact, such bias is more problematic for a study without validation of estimated effects, however, we explicitly validated the estimated effects in the target dataset in the context of polygenic risk prediction.
Ethics oversight	UK Biobank's scientific protocol has been reviewed and approved by the North West Multi-centre Research Ethics Committee (MREC), National Information Governance Board for Health & Social Care (NIGB), and Community Health Index Advisory Group (CHIAG). UK Biobank has obtained informed consent from all participants. Research Ethics approval was obtained from University of South Australia Human Research Ethics Committee (HREC).

Note that full information on the approval of the study protocol must also be provided in the manuscript.
